# Biological Variability and Biomedical Innovation in Personalized Orthodontics: A Systematic Review with Narrative Synthesis

**DOI:** 10.3390/jcm15207767

**Published:** 2026-10-09

**Authors:** Salvatore La Rosa, Rosalia Leonardi, Vincenzo D’Antò, Rosalia Ragusa, Gaetano Isola, Alessandro Polizzi

**Affiliations:** 1 Department of General Surgery and Surgical-Medical Specialties, School of Dentistry, University of Catania, Via S. Sofia 78, 95124 Catania, Italy; salvo.larosa11@live.it (S.L.R.); rleonard@unict.it (R.L.); gaetano.isola@unict.it (G.I.); 2 Department of Neurosciences, Reproductive Sciences and Oral Sciences, School of Orthodontics, University of Naples Federico II, Via Pansini 5, 80131 Naples, Italy; vincenzo.danto@unina.it; 3 Unit of Internationalization, Innovation and Health Research, University Hospital “G. Rodolico–San Marco”, Via Santa Sofia 78, 95123 Catania, Italy; ragusar@unict.it

**Keywords:** personalized orthodontics, orthodontic tooth movement, mechanobiology, biomarkers, genomics, epigenetics, multi-omics, artificial intelligence, precision medicine

## Abstract

**Background/Objectives:** Orthodontic treatment response varies considerably among patients because mechanical loading interacts with individual biological, anatomical, systemic, and behavioral characteristics. Advances in mechanobiology, molecular biomarkers, omics technologies, and artificial intelligence (AI) may support a more personalized approach to treatment. This systematic review (SR) aimed to evaluate human clinical evidence concerning these domains and their potential contribution to predicting orthodontic response, improving clinical decision-making, and reducing adverse effects. **Methods:** A systematic search of PubMed, Scopus, and Web of Science was conducted through June 2026. Primary studies involving human participants and addressing orthodontic tooth movement (OTM), biological variability, biomarkers, genomics, epigenetics, multi-omics, or AI-supported orthodontics were selected according to predefined eligibility criteria. The review was conducted and reported in accordance with Preferred Reporting Items for Systematic Reviews and Meta-Analyses (PRISMA) 2020, and heterogeneous evidence was synthesized narratively across four predefined domains. The review protocol was registered in the International Prospective Register of Systematic Reviews (PROSPERO; registration number CRD420261486348). Risk of bias was assessed using design-appropriate instruments. Certainty of evidence was assessed at the outcome level using GRADE principles adapted to narrative synthesis. **Results:** The search identified 1051 records, of which 14 primary human studies were included. The clinical evidence comprised randomized trials, longitudinal and cross-sectional biomarker studies, genetic association studies, and a case report. Molecular and multi-omics studies identified candidate markers of biological response and patient phenotype. The included computational studies examined genetic association rules, gingival-enlargement classification, and AI-assisted root-resorption measurement; none demonstrated AI-guided selection of orthodontic forces. However, small samples, methodological heterogeneity, limited adjustment for confounding, and lack of external validation restricted clinical translation. Certainty of evidence supporting clinical personalization applications was predominantly very low. **Conclusions:** Personalized orthodontics should remain grounded in comprehensive diagnosis, biologically compatible mechanics, and individualized monitoring. Molecular and computational tools may complement clinical assessment, but routine implementation requires standardized protocols, multicenter validation, and prospective evidence of patient-relevant benefit.

## 1. Introduction

Traditionally, orthodontics has been regarded as the clinical discipline dedicated to the diagnosis, treatment, and prevention of craniofacial and dental malocclusions. However, orthodontic treatment is fundamentally a biological process that extends beyond the mechanical action of appliances. Orthodontic therapy does not merely displace teeth; rather, it induces a programmed and coordinated tissue response involving mechanosensing, mechanotransduction, periodontal ligament adaptation, and alveolar bone remodeling.

Advances in biomedical sciences have improved our understanding of the sequence of cellular and tissue events involved in orthodontic tooth movement (OTM). The periodontal ligament (PDL) and alveolar bone behave as an integrated functional unit in which mechanical strain is translated into biochemical signaling, inflammatory activity, extracellular matrix turnover, and coordinated osteoclastic and osteoblastic responses. Consequently, bone resorption and apposition are not simple passive consequences of loading but components of a tightly regulated biological process. This perspective is becoming increasingly relevant to patient-centered diagnosis and treatment planning [1].

At the same time, modern orthodontics increasingly recognizes that the biological response to treatment is not identical in every patient. Similar force systems may generate different rates of tooth movement, degrees of tissue remodeling, levels of discomfort, and susceptibility to adverse effects. Age, bone metabolism and morphology, local inflammation, treatment duration, movement type, apical displacement, periodontal status, and genetic or epigenetic background may all contribute to this variability. For this reason, avoiding heavy continuous forces and extensive apical displacement over prolonged treatment periods has been recommended [2]. Moreover, malocclusions and dentofacial deformities are now understood as complex, polygenic, and multifactorial traits whose clinical expression and treatment response reflect the interaction between genomic variation and environmental influences [3]. This awareness is shifting orthodontics from a predominantly standardized mechanical approach toward a more individualized biomedical discipline in which treatment is tailored to both the malocclusion and the patient’s biological profile.

Monitoring the biological response during treatment represents a further dimension of personalization. Gingival crevicular fluid (GCF) and saliva are non-invasive, repeatable sources of molecules associated with inflammation, extracellular matrix turnover, osteoclastogenesis, and bone formation. Cytokines and regulatory proteins such as Interleukin-1β (IL-1β), tumor necrosis factor-α (TNF-α), RANKL, osteoprotegerin (OPG), matrix metalloproteinases (MMPs), and alkaline phosphatase can help characterize the temporal phases and interindividual variability of orthodontic remodeling [4,5]. Mechanosensitive microRNAs (miRNAs), long noncoding RNAs (lncRNAs), and circular RNAs (circRNAs) add another regulatory layer by acting at transcriptional, post-transcriptional, and epigenetic levels, with possible future roles as biomarkers or therapeutic targets [6]. Mechanical loading can also modify DNA methylation, histone marks, chromatin accessibility, and metabolic–epigenetic signaling, potentially creating a persistent cellular “mechanical memory” [7]. None of these approaches is ready for routine orthodontic use, but together they broaden the biological basis on which precision-oriented care may eventually develop.

The same concept extends to artificial intelligence, which offers a way to combine imaging, clinical variables, biological markers, and longitudinal treatment information within a common analytical framework. Artificial intelligence (AI) and machine learning (ML) are already being investigated for automated image analysis, growth assessment, risk stratification, treatment simulation, outcome prediction, and remote monitoring. Their potential contribution is not simply automation, but the ability to detect clinically relevant patterns across variables that are difficult to interpret in isolation. Performance, however, is uneven across applications, and clinician supervision remains necessary. Responsible implementation therefore depends on transparent methods, external validation, representative datasets, appropriate governance, privacy protection, and safeguards against algorithmic bias [8,9].

From this perspective, emerging biomedical sciences and AI should be interpreted as complementary instruments that refine, rather than replace, orthodontic reasoning. Their integration with biomechanics and clinical expertise may progressively enable care that is more biologically informed, predictive, preventive, and patient-specific. Therefore, this systematic review (SR) aimed to examine the biological foundations of OTM, the determinants of individual variability, and the emerging contribution of biomarkers, genomics, epigenetics, other omics technologies, and artificial intelligence to personalized orthodontics, using a narrative synthesis appropriate to the heterogeneity of the available evidence.

## 2. Materials and Methods

This SR used a structured and transparent process for study identification, selection, data extraction, and evidence synthesis. The review was conducted and reported in accordance with the Preferred Reporting Items for Systematic Reviews and Meta-Analyses (PRISMA) 2020 statement. The completed PRISMA 2020 checklist is provided as Supplementary Table S1 [10]. Because substantial heterogeneity was anticipated across clinical, translational, experimental, molecular, and computational studies, the findings were synthesized narratively rather than quantitatively.

### 2.1. Protocol and Registration

The review protocol was registered in the International Prospective Register of Systematic Reviews (PROSPERO; registration number CRD420261486348). The systematic evidence set was restricted to primary human studies; non-human and secondary sources were retained only for context. Database-search results were unchanged.

### 2.2. Research Question

The review addressed the following research question: “How do patient-specific biological, molecular, genetic, craniofacial, and computational characteristics contribute to the characterization, prediction, or monitoring of orthodontically relevant phenotypes, treatment response, and adverse effects, and what is their current relevance to personalized orthodontic care?”

Within this framework, direct evidence of personalization was defined as evidence concerning individual treatment response, treatment-related biological changes, adverse effects, longitudinal monitoring, or predictive performance. Evidence concerning pretreatment genetic, molecular, dental, or craniofacial phenotypes was considered enabling evidence for patient stratification and was interpreted separately from direct evidence of treatment personalization.

### 2.3. Justification

Individual variability significantly influences orthodontic treatment response and the risk of adverse effects. However, evidence concerning mechanobiology, biomarkers, genomics, epigenetics, and AI remains fragmented. This SR integrates these fields to assess their contribution to personalized orthodontics and identify current limitations and future research priorities. Accordingly, study identification, selection, extraction, and reporting followed a predefined systematic methodology, while narrative synthesis was used to accommodate methodological and outcome heterogeneity.

### 2.4. Eligibility Criteria

Studies were eligible if they: (1) were primary investigations involving human participants; (2) examined orthodontic treatment, orthodontic tooth movement, malocclusion, or treatment-related biological variability; (3) evaluated clinically relevant mechanobiological variables, biomarkers, genomics, epigenetics, multi-omics, microbiome-related profiles, or AI-supported personalization; (4) reported patient-level clinical, biological, diagnostic, predictive, or safety outcomes; and (5) were peer-reviewed full-text articles published in English. Eligible designs comprised randomized and non-randomized clinical studies, prospective or retrospective cohort studies, case–control studies, cross-sectional studies, longitudinal biomarker investigations, and clinically informative case reports. Reviews, systematic reviews, book chapters, editorials, conference proceedings, interviews, conceptual reports, animal studies, in vitro investigations, finite element analyses, and studies without original human data were excluded from the systematic evidence set. These sources could be cited only to provide biological or technological context in the Introduction and Discussion and were not included in the PRISMA count, study-characteristics table, or risk-of-bias assessment.

Moreover, eligible studies were classified according to their relationship with personalized care. Direct personalization studies evaluated interindividual differences in orthodontic treatment response, tooth movement, treatment-related biological changes, adverse effects, monitoring, or diagnostic or predictive performance. Enabling patient-stratification studies evaluated patient-specific genetic, molecular, dental, or craniofacial characteristics that could contribute to baseline phenotype or susceptibility assessment but did not directly test treatment response. The latter were retained as upstream evidence relevant to personalized assessment but were not interpreted as demonstrating treatment prediction or treatment-selection utility.

The PICO framework comprised patients undergoing orthodontic assessment or treatment and human participants investigated for orthodontically relevant biological or craniofacial characteristics (Population); biomarker, genomic, epigenetic, multi-omics, mechanobiological, or AI-supported assessment and personalization strategies (Intervention/Exposure); conventional clinical assessment, untreated or differently treated controls, contralateral control sites, baseline measurements, or alternative analytical methods, where applicable (Comparison); and patient-specific craniofacial or dental phenotype and biological susceptibility for enabling patient stratification, or individual treatment response, tooth movement, treatment duration, pain, root resorption, periodontal changes, treatment-related molecular variation, monitoring performance, and clinical decision support for direct personalization evidence (Outcome).

For eligibility purposes, biological variability included between-patient or longitudinal differences in orthodontically relevant clinical or biological measurements; biomarkers were measurable molecular, cellular, or tissue characteristics obtained from human participants. Multi-omics involved the integration of at least two molecular data types, while AI-supported studies used computational methods for orthodontically relevant classification, prediction, measurement, or monitoring; measurement alone was not interpreted as prediction. Studies combining human and non-human experiments were eligible only if the human results were separately extractable; primarily mechanistic studies were eligible when they reported orthodontically relevant measurements in human participants.

### 2.5. Literature Sources and Search Parameters

The final searches of PubMed, Scopus, and Web of Science were conducted on 27 June 2026. In the Web of Science platform, the “All Databases” search option was selected rather than Web of Science Core Collection alone. A health sciences librarian assisted in developing a search strategy that incorporated all identified keywords and free-text terms. The Web of Science, Scopus, and PubMed databases were used. In addition, the reference lists of the included articles were examined to identify further potentially relevant evidence. The search strategy was adapted to each database, as presented in Table 1.

The search was deliberately focused on studies linking orthodontics with patient-specific care and biological or computational approaches. It was not designed to retrieve the entire literature on orthodontic mechanobiology or omics independently of its relevance to personalization.

PubMed was selected for biomedical and clinical studies, Scopus for its broad multidisciplinary coverage, and Web of Science All Databases for complementary citation indexing across scientific disciplines. The complete database-specific queries, including field tags, the PubMed MeSH term, free-text terms, and Boolean operators, are reproduced in Table 1. Reference lists of the included studies were screened for additional eligible reports. IEEE Xplore and Embase were not searched, and no dedicated grey-literature search was conducted; relevant computational or biomedical studies indexed only in those sources may therefore have been missed. No language, publication-date, or study-design filters were applied to the database searches; the English-language and publication-type eligibility criteria were applied during screening.

### 2.6. Data Management

#### 2.6.1. Study Selection

Following retrieval from each electronic database, all citations were imported into EndNote 20 reference-management software (Clarivate, London, UK). Duplicate records were identified using EndNote 20 and then checked manually to confirm matching citations and resolve records not recognized automatically. Reports presenting overlapping findings from the same study were also reviewed manually to avoid counting the same study twice. Titles and abstracts were screened independently by S.L.R. and A.P. according to the predefined eligibility criteria. Potentially eligible reports underwent independent full-text assessment by the same two reviewers, S.L.R. and A.P. Only primary studies reporting original data from human participants were included in the systematic review. Reviews, book chapters, preclinical models, in vitro studies, finite element analyses, conceptual reports, and other non-eligible publication types were excluded from the systematic evidence set, although relevant sources could subsequently be used for contextual interpretation. Disagreements were resolved through discussion or consultation with R.L. Agreement between the two reviewers was assessed using Cohen’s kappa statistic (k = 0.89).

#### 2.6.2. Data Extraction

Two reviewers, S.L.R. and A.P., developed a standardized data-extraction form to collect the study characteristics and outcomes required for the subsequent analysis, including study design, sample size, objectives, and principal findings.

Relevant data were extracted independently by S.L.R. and A.P. from each included human study, including publication year, country, study design, participant characteristics, sample size, orthodontic intervention or exposure, comparator, biological specimen or data source, follow-up, primary outcomes, principal findings, and information required for risk-of-bias assessment. Clinical relevance, methodological limitations, and potential applications in personalized orthodontics were also recorded. Discrepancies were resolved by discussion with R.L. Agreement between the two reviewers was assessed using Cohen’s kappa statistic (k = 0.89).

The outcomes sought included the rate and extent of orthodontic tooth movement; treatment-related biological and molecular changes; biomarker concentrations; genomic, epigenetic, and multi-omics profiles; pain; root resorption; periodontal changes; craniofacial phenotype; and the diagnostic or predictive performance of AI-supported methods. All relevant outcomes, assessment methods, and time points reported by each included study were considered.

### 2.7. Risk-of-Bias Assessment

Risk of bias was evaluated for all 14 included human studies using design-appropriate tools. The three randomized clinical trials were assessed using the revised Cochrane risk-of-bias tool (RoB 2) [11] across five domains: randomization, deviations from intended interventions, missing outcome data, outcome measurement, and selection of the reported result. Domain and overall judgments followed the RoB 2 algorithm. Three case–control studies and one comparative cohort study were evaluated using the appropriate Newcastle–Ottawa Scale (NOS) [12] forms, with item-level stars recorded for selection, comparability, and exposure or outcome ascertainment. Four single-group before–after studies were assessed using the JBI [13] quasi-experimental checklist. Two analytical cross-sectional studies and one case report were assessed using the corresponding JBI checklists. JBI responses were recorded item by item as Yes, No, Unclear, or Not applicable. No numerical cutoffs were used to convert NOS stars or JBI responses into overall low-, moderate-, or high-risk categories. S.L.R. and A.P. performed independently and agreed on the revised item- and domain-level judgments reported in Supplementary Table S2; any disagreements were resolved by discussion or consultation with R.L. The item- and domain-level appraisal, together with supporting reasons, is presented in Supplementary Table S2. Risk of bias due to missing results and publication bias could not be formally assessed because the studies were few and did not form sufficiently comparable outcome groups. Possible publication bias was considered qualitatively when interpreting certainty, but its presence or absence could not be established. Certainty of evidence was assessed for the principal outcome-level conclusions using GRADE principles adapted to narrative synthesis, following Murad et al. [14]. Risk of bias, inconsistency, indirectness, imprecision, and publication bias were considered. Where only one study addressed an outcome, between-study inconsistency could not be assessed. Certainty judgments and their supporting reasons are reported in Supplementary Table S3 [14,15,16,17,18,19,20,21,22,23,24,25,26,27,28]. No single certainty rating was assigned to an entire thematic domain when its studies addressed different outcomes.

### 2.8. Information Synthesis

The findings from the 14 included human studies were synthesized narratively because study designs, populations, exposures or interventions, biological specimens, analytical methods, and outcomes differed substantially. Evidence was organized into four predefined domains: Biological Foundations of Orthodontic Tooth Movement; Biological Variability and Individual Response; Emerging Biomedical Sciences in Orthodontics (biomarkers, genomics, and epigenetics); and the Role of Artificial Intelligence in Personalized Orthodontics. The four-domain framework was specified before title and abstract screening and data extraction; studies were subsequently assigned to these domains according to their research question and measured outcomes. Mechanistic reviews, animal studies, in vitro investigations, and conceptual publications were used only to contextualize the human findings in the Discussion and were not treated as included studies.

Quantitative pooling was not undertaken because the included human studies differed substantially in design, participant characteristics, orthodontic intervention or exposure, sampling schedule, molecular or computational method, and outcome definition. A structured narrative synthesis was therefore considered the most appropriate method for comparing findings, identifying consistent patterns, and highlighting clinical and translational gaps.

Within each domain, studies were further distinguished as providing either direct personalization evidence or enabling patient-stratification evidence. Evidence concerning craniofacial or dental phenotypes without evaluation of orthodontic treatment response was synthesized separately and was not considered evidence of treatment-effect prediction or clinically validated treatment personalization.

For interpretation, the included evidence was further distinguished by the clinical question addressed: biological mechanisms underlying interindividual variability; measurement or prediction of the rate or magnitude of tooth movement; prediction of treatment duration or overall treatment outcomes; assessment or prediction of adverse effects; and AI-assisted measurement, monitoring, or decision support. Observed molecular changes, associations with pretreatment phenotypes, and methods used to quantify outcomes were not considered evidence of individual-level prediction unless predictive performance for the relevant clinical outcome had been evaluated. Domains for which no included study directly tested a predictive or treatment-selection claim were identified as evidence gaps.

The narrative synthesis did not assign equal evidentiary weight to all study designs. Findings were interpreted in light of the presence of a comparator, sample size, outcome relevance, design-specific risk of bias, and outcome-level certainty. The single case report was used only to describe a possible phenotype–variant observation; it was not treated as evidence of an association, predictive performance, or treatment effect. Because the studies addressed different clinical questions and did not yield comparable effect measures for a common outcome, a pooled estimate would not have been clinically interpretable.

Outcomes were also distinguished as surrogate biological or analytical measures (such as biomarker concentrations, molecular profiles, genetic associations, and automated measurements) or patient-relevant clinical outcomes (such as treatment duration and quality, adverse events, patient burden, and treatment predictability). Evidence concerning a surrogate measure was not interpreted as evidence of clinical benefit unless its effect on a patient-relevant outcome had been evaluated.

## 3. Results

### 3.1. Identified Studies

**Figure 1:**
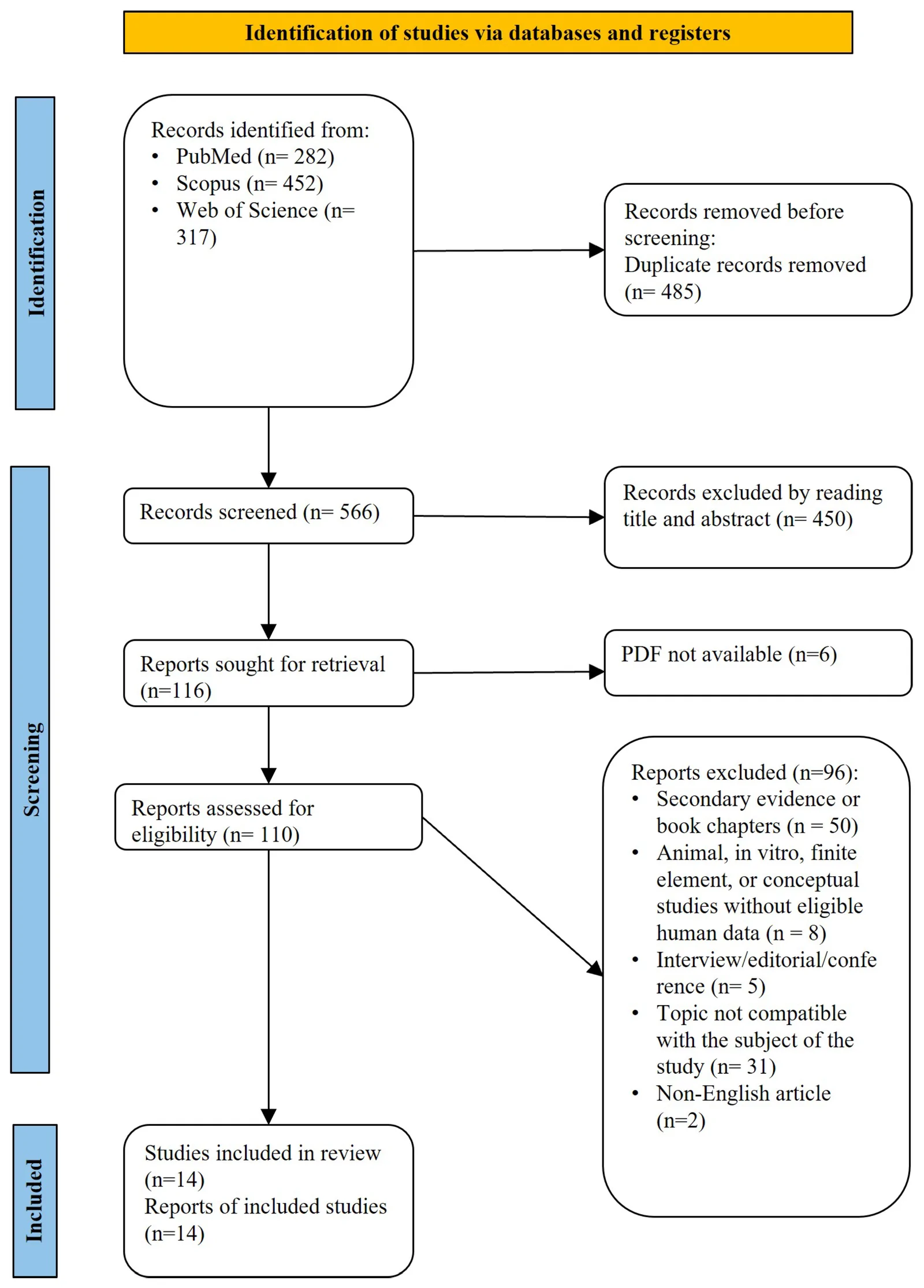
PRISMA 2020 flow diagram of study identification, screening, eligibility assessment, and inclusion of primary human studies.

The database search yielded 1051 records: 282 from PubMed, 452 from Scopus, and 317 from Web of Science. After removal of 485 duplicates, 566 records underwent title and abstract screening, and 450 were excluded. Full texts were sought for the remaining 116 reports; six could not be retrieved. The full texts of 110 reports were assessed for eligibility. Of these, 96 were excluded: 50 were secondary evidence or book chapters, eight were animal, in vitro, finite element, or conceptual investigations without eligible original human data, 31 were incompatible with the review question, five were editorials, interviews, or conference publications, and two were not published in English. Fourteen primary human studies met the eligibility criteria and were included in the systematic review and risk-of-bias assessment. The study-selection process is shown in Figure 1. The list of articles excluded is in Supplementary Table S4 [1,2,3,4,5,6,7,9,29,30,31,32,33,34,35,36,37,38,39,40,41,42,43,44,45,46,47,48,49,50,51,52,53,54,55,56,57,58,59,60,61,62,63,64,65,66,67,68,69,70,71,72,73,74,75,76,77,78,79,80,81,82,83,84,85,86,87,88,89,90,91,92,93,94,95,96,97,98,99,100,101,102,103,104,105,106,107,108,109,110,111,112,113,114,115,116].

### 3.2. Summary of Identified Studies

All 14 included human studies underwent design-specific critical appraisal. The three randomized trials received an overall RoB 2 judgment of some concerns. Four comparative observational studies were assessed with the NOS [16,25,27,28]. Four single-group before–after studies, two analytical cross-sectional studies, and one case report were assessed with the corresponding JBI checklists. The item-level assessments identified recurring concerns regarding the absence of independent controls, incomplete control of confounding, and uncertainty about some analytical choices. No overall risk category was inferred from NOS or JBI totals. Individual judgments and explanations are reported in Supplementary Table S2. Sample sizes ranged from a single clinically characterized patient to 152 tissue samples, and the studies investigated cytokine responses, periodontal and salivary biomarkers, microRNAs, metabolomics, microbiome profiles, genetic variants, root resorption, and AI-assisted or algorithmic prediction. Considerable heterogeneity was observed in participant selection, orthodontic interventions, sampling schedules, biological specimens, analytical platforms, follow-up periods, and outcome definitions. The principal characteristics and findings of the included human studies are summarized in Table 2.

Because the included studies investigated heterogeneous outcomes using different analytical methods, the findings were presented descriptively, and a common effect estimate was not applicable.

### 3.3. Thematic Distribution of the Evidence

The findings from the included studies were organized into four domains to facilitate interpretation and provide a clearer overview of the available evidence: Biological Foundations of Orthodontic Tooth Movement; Biological Variability and Individual Response; Emerging Biomedical Sciences in Orthodontics (biomarkers, genomics, and epigenetics); and the Role of Artificial Intelligence in Personalized Orthodontics.

Ten studies [15,16,17,18,19,20,21,22,23,24] provided direct evidence concerning treatment-related biological response, monitoring, adverse-effect assessment, or analytical prediction, whereas four studies [25,26,27,28] evaluated genetic or molecular associations with hypodontia, mandibular retrognathism, open bite, or complex craniofacial phenotypes. The latter studies were classified as enabling patient-stratification evidence. Although potentially relevant to pretreatment characterization, they did not evaluate orthodontic treatment response and therefore do not establish predictive or treatment-selection utility.

The biological-foundations domain included clinical investigations of early cytokine responses, periodontal gene expression, force-related microRNA regulation, and tooth-movement-associated remodeling [16,19,21]. Biological variability and individual response were represented by studies of personalized force regimens, craniofacial and dental phenotypes, and genetic associations [18,25,26,27,28]. Emerging biomedical sciences constituted the largest domain and included cytokine analysis, genomics, microRNAs, spectroscopy, metabolomics, microbiome profiling, and biomarker-based risk stratification [15,16,17,19,20,21,22,23,24,25,26,27,28]. Among the included studies, computational methods were used for genetic association-rule analysis [25], gingival-enlargement classification [20], and AI-assisted measurement of root resorption [18]. These studies addressed different clinical or analytical tasks and did not demonstrate AI-guided force selection. The methodological characteristics, validation strategies, performance metrics, and clinical evaluation of the computational approaches identified among the included studies are summarized in Table 3.

### 3.4. Results of Individual Studies

The included studies addressed different stages of patient characterization and orthodontic care. Their principal observed results are reported below by evidence domain. Statistical associations and differences in molecular measurements are distinguished from demonstrated treatment prediction or clinical benefit.

Biological response to orthodontic treatment. In 20 patients, Kumar et al. [16] observed an increase in gingival crevicular fluid IL-1 from 15.67 to 27.94 pg/L following force activation (*p* = 0.009). The corresponding concentration at the post-activation assessment was higher in adolescents than in young adults (42.96 versus 17.93 pg/L; *p* = 0.006). In the randomized study by Spitz et al. [21], 15 participants underwent tooth movement either alone (*n* = 7) or with micro-osteoperforations (*n* = 8). Compared with unloaded contralateral teeth, 331 genes were differentially expressed after tooth movement alone and 356 after tooth movement with micro-osteoperforations. No genes met the prespecified differential-expression threshold in the direct comparison between the two treatment groups. Polizzi et al. [19] studied 21 patients in a split-mouth canine-distalization design and observed changes in gingival crevicular fluid microRNAs over three months. Baseline miRNA-7a-2-3p (*p* = 0.025), miRNA-21-5p (*p* = 0.014), miRNA-200b-3p (*p* = 0.041), miRNA-125b-2-3p (*p* = 0.021), age (*p* = 0.042), and bleeding on probing (*p* = 0.021) were associated with the amount of tooth movement in the reported multivariable model. These analyses did not establish externally validated predictive performance.

Pretreatment phenotypes and genetic variation. In the case–control study by Paddenberg-Schubert et al. [27], the EGF rs4444903 minor allele and the EGF rs4444903|rs2237051 haplotype were more frequent among participants with an orthognathic mandible than among those with mandibular retrognathism (*p* = 0.008 and *p* = 0.007, respectively; 45 controls and 74 cases). Yılmaz et al. [28] found no significant difference in the overall distribution of MMP-1 rs1799750 genotypes or alleles between 30 open-bite patients and 30 controls (*p* = 0.2269 and *p* = 0.7125). A within-case comparison suggested variation in genotype frequency by open-bite severity (*p* = 0.009); however, the case group deviated from Hardy–Weinberg equilibrium (*p* = 0.015), warranting cautious interpretation. Grenga et al. [26] described a single patient with craniofacial and dental anomalies carrying MIR146A rs2910164 and MIR182 rs76481776 variants. The case provides no comparison group or estimate of association or causality. In Alhazmi et al. [25], 106 participants were studied, including 54 with hypodontia and 52 controls. Logistic regression did not identify a statistically significant association with dental impaction (*p* = 0.112). Association-rule analysis identified a PAX9 C/C–MSX1 A/A combination with a lift of 1.671, but this statistic does not demonstrate validated predictive accuracy.

Treatment outcomes and molecular monitoring. Navarro-Fraile et al. [18] reported preliminary results from 18 randomized patients. After 142 days, AI-measured mean root-length loss was 0.20 ± 0.23 mm with the conventional force sequence and 0.42 ± 0.43 mm with the individualized sequence (*p* = 0.045). Corresponding total root-volume losses were 2.44 ± 6.59 and 2.42 ± 4.75 mm^3^ (*p* > 0.05). AI was used for outcome measurement rather than force selection. In the five-patient pilot study by Kapoor et al. [15], salivary and serum miRNA-200a were assessed before and nine months after functional-appliance therapy. Neither the salivary nor the serum change in ΔCt was statistically significant (*p* = 0.686 and *p* = 0.715, respectively). Lepore et al. [17] examined gingival crevicular fluid from six patients at baseline, two days, and two weeks. ATR-FTIR spectroscopy identified treatment-related changes in selected lipid- and protein-associated spectral ratios; the reported change in the 1237/1651 cm^−1^ band-area ratio was significant at *p* < 0.05. The study did not test whether spectral measurements predicted an individual clinical outcome.

Adverse effects and multi-omics profiles. In the cross-sectional analysis by Simancas-Escorcia et al. [20], S100A4-positive fibroblast density was 245.8 ± 38.7 cells/mm^2^ in gingival-enlargement samples, 165.3 ± 29.4 cells/mm^2^ in orthodontic controls, and 98.2 ± 18.5 cells/mm^2^ in healthy controls (*p* < 0.001). The reported adjusted odds ratio was 1.028 per additional cell/mm^2^ (95% CI 1.021–1.035), and the reported discrimination was AUC = 0.952 (95% CI 0.921–0.983). These performance estimates arose from the study dataset and require prospective external validation. Velachery et al. [23] analyzed saliva from six patients before and two weeks after appliance removal. Of 2153 identified metabolites, 320 were detected at both time points, and 50 shared metabolites showed a significant fold change; the small uncontrolled sample limits attribution of these differences to appliance removal. In 61 adolescents, Xie et al. [22] found that oral health status was more strongly associated with variation in microbiota composition than appliance type (ANOSIM *R* = 0.29 versus 0.09; both *p* = 0.001). The metabolomic analysis detected 1977 metabolites and selected 13 as candidate markers associated with poor oral health; clinical predictive performance was not externally validated. Finally, Zhang et al. [24] compared salivary profiles at approximately two and 12 months in 30 orthodontic patients and reported a difference in ApoE expression, supported by Western blotting. The publication did not establish an individual-level prediction threshold or report validated discrimination for treatment response.

Adjustment for potential confounding was limited and varied across studies. Polizzi et al. [19] reported a multivariable analysis including age and bleeding on probing alongside baseline miRNAs, whereas Simancas-Escorcia et al. [17] reported an adjusted association for S100A4-positive fibroblast density. The other reported biomarker and genetic findings were not consistently supported by analyses accounting for clinical, periodontal, behavioral, and treatment-related factors. Accordingly, differences in age, periodontal inflammation, treatment stage, mechanics, appliance type, systemic exposures, or adherence may contribute to some observed associations. The available data do not permit these influences to be separated reliably for every study.

### 3.5. Certainty of Evidence

The certainty of evidence for the clinical application of the investigated molecular, genetic, and computational approaches was predominantly very low (Supplementary Table S3). Small samples, limitations in study design, imprecision, indirectness to clinical decision-making, and lack of external validation contributed to these judgments. The genetic studies investigated craniofacial or dental phenotypes rather than predicting orthodontic treatment response. None of the included studies directly demonstrated that AI-guided force selection improves patient outcomes; certainty could therefore not be rated for that claim.

Across the four domains, risk-of-bias concerns constrained interpretation in different ways: the small randomized studies did not establish robust treatment-selection effects; uncontrolled longitudinal biomarker studies could not reliably separate treatment-related changes from time-dependent or other influences; genetic and cross-sectional associations remained susceptible to confounding and selection bias; and the single case report could not support an association or causal inference. These design-specific limitations informed the outcome-level certainty judgments in Supplementary Table S3.

## 4. Discussion

This SR with narrative synthesis identified 14 primary human studies relevant to personalized orthodontic treatment. The clinical evidence indicates that patient response cannot be explained by force magnitude or appliance design alone and may also reflect biological, molecular, periodontal, genetic, and behavioral variability. Biomarkers, omics approaches, and AI provide potentially useful information for risk assessment and longitudinal monitoring, but the included studies remain heterogeneous and mostly exploratory. Mechanistic reviews, animal studies, in vitro experiments, and conceptual publications are discussed only to contextualize these human findings and should not be interpreted as part of the systematically included evidence. The following sections examine the four predefined domains, emphasizing the distinction between mechanistic plausibility and clinically validated utility.

Importantly, not all included studies evaluated treatment personalization directly. Studies of hypodontia, mandibular retrognathism, open bite, and complex craniofacial phenotypes provide enabling evidence concerning baseline patient characterization rather than evidence that treatment response can be predicted. Their findings should therefore be interpreted as hypothesis-generating inputs for future stratification research.

Clinical translation requires three distinct steps: demonstrating an association between a candidate biomarker and an orthodontic phenotype or response; showing that the biomarker improves individual-level prediction beyond conventional clinical variables in externally validated models; and demonstrating that biomarker-guided decisions improve patient-relevant outcomes. The included studies primarily addressed the first step or described treatment-related molecular changes. They did not establish incremental predictive value over conventional assessment or clinical benefit from biomarker-guided treatment. These findings should therefore be considered preliminary and hypothesis-generating.

### 4.1. Biological Foundations of Orthodontic Tooth Movement

#### 4.1.1. From Orthodontic Force to Periodontal Strain

OTM is an aseptic remodeling process involving the tooth, PDL, alveolar bone, cementum, vasculature, and neural components. Appliance-generated forces are defined by their magnitude, direction, duration, point of application, and moment-to-force ratio; together with the relationship between the line of action and the center of resistance, these variables determine movement and periodontal stress distribution [105].

Force application initially displaces the tooth within its socket and deforms the viscoelastic PDL [1]. Although compression and tension remain useful clinical concepts, stress is distributed heterogeneously according to root morphology, alveolar anatomy, PDL thickness, and movement type. Finite element studies confirm movement-specific stress concentrations, but their translation to human OTM remains limited because computational, cellular, and animal models reproduce only selected features of the periodontal environment [55,68].

#### 4.1.2. Mechanosensing and Mechanotransduction

PDL fibroblasts, osteoblast-lineage cells, osteocytes, endothelial and immune cells, and sensory nerve endings detect extracellular-matrix deformation and altered fluid flow. Integrins, the cytoskeleton, mechanosensitive ion channels, primary cilia, and cell junctions convert these stimuli into signaling through focal adhesion kinase, MAPK/ERK, Wnt/β-catenin, prostaglandins, nitric oxide, and hypoxia-responsive pathways. The PDL and alveolar bone therefore function as an integrated mechanosensitive unit [1,107].

Compression transiently reduces local perfusion and oxygen tension, whereas tensile strain modifies vascular permeability and angiogenic activity. The resulting release of prostaglandin E2, nitric oxide, growth factors, neuropeptides, and cytokines—including IL-1β, IL-6, IL-17, and TNF-α—initiates the sterile inflammatory response required for remodeling [4,90]. The early increase in GCF IL-1β after force activation supports the transient nature of this response [16].

#### 4.1.3. Osteoclastogenesis, Osteogenesis, and Extracellular Matrix Remodeling

Bone resorption is principally regulated by the RANKL/OPG system. Under compression and hypoxia, PDL and bone cells increase RANKL and macrophage colony-stimulating factor, promoting osteoclast differentiation, whereas OPG limits this process. TNF-α, IL-1β, prostaglandins, and matrix metalloproteinases reinforce catabolic activity and extracellular-matrix turnover [1,4]. Human GCF proteomics similarly indicates a temporally ordered progression from inflammation and resorption to repair [5].

At sites predominantly exposed to positive strain, Wnt-related pathways and RUNX2 favor osteoblast differentiation, matrix production, and mineralization, while osteocytes coordinate resorptive and formative activity [1,107]. Transcriptomic evidence confirms that loading modifies networks related to bone metabolism, inflammation, and immune regulation [21]. OTM therefore depends on coupled resorption and formation. Notably, donor-related differences in PDL stromal-cell gene expression may exceed the effect of mechanical strain itself, providing a molecular basis for heterogeneous responses to comparable loading [72].

#### 4.1.4. Temporal Phases and the Biological Importance of Force Magnitude

Clinically, OTM is often described as an initial displacement phase, a lag phase, and a post-lag or progressive phase. Initial movement reflects PDL deformation and fluid displacement. If compression exceeds the adaptive capacity of the ligament, blood flow may be interrupted and localized sterile necrosis, historically termed hyalinization, may occur. Tooth movement then slows while macrophages remove the necrotic tissue and osteoclasts resorb bone from adjacent marrow spaces through undermining resorption. Once the damaged area has been cleared, frontal resorption and progressive movement can resume [1].

This sequence explains why increasing force magnitude does not necessarily produce faster or more efficient movement. Excessive forces can enlarge hyalinized areas, prolong the lag phase, intensify pain, and increase the risk of root resorption and periodontal damage. In contrast, low-magnitude continuous forces are more likely to preserve tissue perfusion and support coordinated modeling and remodeling of the alveolar process [109]. The definition of an optimal force remains controversial because movement rate depends not only on magnitude but also on force duration, root surface and morphology, movement type, bone density, PDL turnover, and individual tissue response [81].

The conventional pressure–tension model is complemented by evidence of a broader early catabolic response followed by a generalized anabolic phase, suggesting temporal coordination across the root rather than strictly separated biological compartments [37]. Circadian variation in osteogenic-factor expression further indicates that the timing of force application may influence remodeling efficiency [99]. These shared mechanobiological pathways provide the general framework of OTM; however, their magnitude, timing, and clinical expression vary considerably among patients, making individual biological variability the next essential level of analysis.

**Figure 2:**
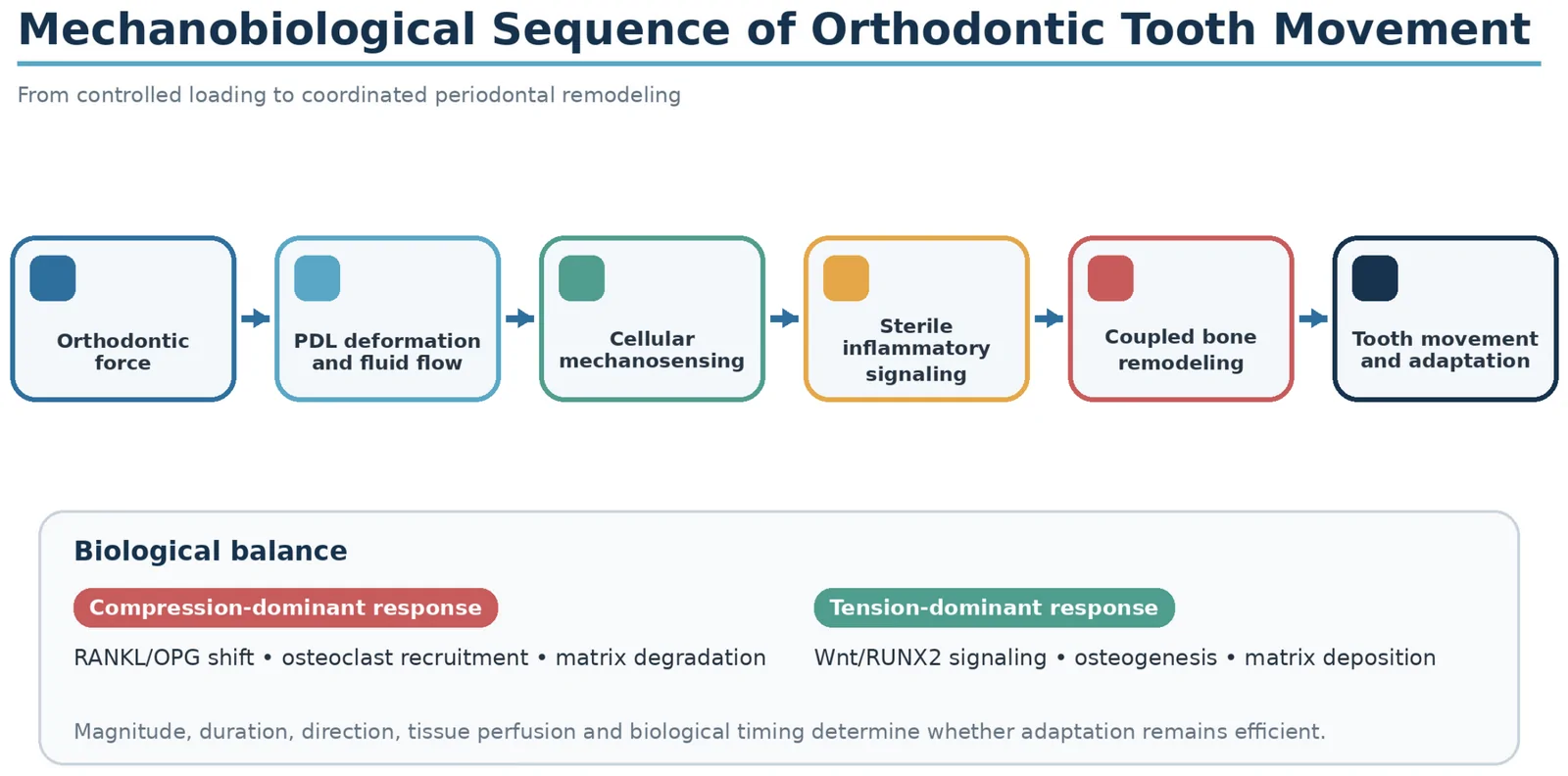
Conceptual sequence linking orthodontic force application to periodontal mechanosensing, inflammatory signaling, coupled bone remodeling, and tooth movement.

The integrated mechanobiological sequence underlying these events is summarized in Figure 2.

### 4.2. Biological Variability and Individual Response

#### 4.2.1. A Multifactorial Response to Orthodontic Loading

Orthodontic mechanics do not produce an identical biological response in every patient. With comparable appliances and nominal force levels, patients may differ in the rate and pattern of tooth movement, duration of the lag phase, inflammatory response, pain, periodontal adaptation, root resorption, and post-treatment stability. This variability reflects the interaction between the applied force system and a patient-specific biological context that includes age, skeletal maturity, periodontal and alveolar anatomy, systemic health, medication use, genetic background, lifestyle, and psychosocial factors. Personalized orthodontics therefore requires stratification of patients according to clinically meaningful subgroups rather than the assumption that one force prescription is universally optimal [71]. Direct experimental support for this variability comes from primary human PDLSCs exposed to the same tensile-loading protocol: donor-dependent transcriptomic differences were substantially greater than the force-related expression changes, demonstrating that a standardized mechanical stimulus can generate markedly different molecular responses [72].

Individual response is also dynamic. The same patient may show different tissue behavior at different treatment stages because inflammatory activity, vascular perfusion, bone turnover, appliance activation, adherence, and local anatomy change over time. Personalized planning consequently involves repeated assessment and adaptation, not merely a single pretreatment classification. This broader model combines biological characteristics with environment and lifestyle, while also incorporating customized appliances and biomechanical delivery [73].

Personalization, however, should not be equated with adding unvalidated biological variables to routine care. A predictor becomes clinically useful only if it is reproducible, contributes information beyond conventional examination, and changes treatment or monitoring in a way that improves outcomes. At present, individualized clinical assessment remains more dependable than molecular prediction when these conditions have not been demonstrated [83].

#### 4.2.2. Age, Biological Timing, Sex, and Pain Perception

Age influences OTM through changes in PDL cellularity, vascularity, bone density, immune signaling, and remodeling capacity. Younger patients generally exhibit more active bone turnover, whereas adults may show slower cellular recruitment and a longer remodeling response; chronological age alone, however, is an imprecise surrogate for biological age. Skeletal maturity, endocrine status, local bone characteristics, and systemic condition are therefore more informative when considered together. Circadian oscillation of osteogenic mediators (with the night more efficient than the day) also suggests that tissue responsiveness varies with biological timing, although the evidence is not sufficient to define clinical activation schedules [99]. A combined analysis of human CT data and age-stratified animal models further indicated that jawbone density may modify the response to non-invasive acceleration strategies. Low-frequency vibration was more effective in lower-density adolescent models, whereas high-frequency vibration produced greater acceleration in denser young-adult models; adding near-infrared light produced a further synergistic effect. These observations are consistent with the concept of density-adapted stimulation, but their clinical relevance still rests largely on preclinical evidence [117].

Pain provides a clear clinical example of interindividual variability. Orthodontic pain typically begins within hours of force application, peaks during the first day, and decreases over the following days, but its intensity is shaped by age, sex-age interactions, anxiety, previous pain experience, inflammatory status, treatment modality, and psychosocial context. Because discomfort can reduce adherence or lead to treatment discontinuation, patients with a high-risk pain phenotype may benefit from anticipatory counseling and individualized non-pharmacological support. Moderate-certainty evidence currently favors low-level laser therapy and chewing interventions, whereas evidence for vibration and psychological strategies is less certain [112].

#### 4.2.3. Systemic Health, Medications, and Lifestyle

Systemic diseases can modify orthodontic remodeling by altering inflammation, angiogenesis, immune function, and osteoblast-osteoclast coupling. Diabetes is particularly relevant: poor glycemic control and accumulation of advanced glycation end-products may impair osteogenic differentiation and wound healing while amplifying periodontal inflammation. Chronic inflammatory disorders, endocrine abnormalities, osteoporosis, and disturbances of vitamin D or thyroid metabolism may likewise change the expected response. Evidence from dental stem-cell models supports the concept that hyperglycemic, inflammatory, microbial, and molecular conditions can alter progenitor-cell viability and differentiation, although direct clinical translation remains limited [106].

A complete medication history is therefore integral to orthodontic diagnosis. Nonsteroidal anti-inflammatory drugs (NSAIDs), bisphosphonates, corticosteroids, and selected hormonal therapies may reduce osteoclastic activity and slow movement, whereas prostaglandins, vitamin D metabolites, thyroid hormones, and other molecular modulators have shown acceleration effects, predominantly in preclinical models. The direction and magnitude of the effect depend on dose, duration, route of administration, systemic condition, and the phase of remodeling. Current evidence does not justify pharmacological manipulation solely to accelerate routine treatment, but it does support adapting mechanics and monitoring when patients already receive agents that affect bone metabolism [94]. A recent SR similarly found clinically relevant effects for NSAIDs and antiresorptive agents but rated the overall certainty of evidence as low to very low [58].

Lifestyle and oral-environmental factors add another layer of variability. Fixed and removable appliances create different plaque-retentive surfaces and may disturb the oral microbial equilibrium. The clinical consequences depend strongly on oral hygiene, diet, salivary characteristics, smoking, appliance design, and adherence. Dysbiosis may increase gingival inflammation, enamel demineralization, caries, and periodontal disease; meticulous mechanical plaque control remains the most important preventive measure [118].

#### 4.2.4. Periodontal and Dentoalveolar Phenotype

The periodontal phenotype defines the anatomical boundary within which tooth movement can be performed safely. Thin gingival tissues, reduced cortical thickness, pre-existing dehiscence or fenestration, diminished alveolar support, and active periodontal inflammation can increase susceptibility to recession, bone loss, and root exposure. Thick phenotypes generally provide a broader adaptive envelope, but they do not eliminate risk when displacement exceeds the alveolar housing. Force magnitude and direction, movement type, periodontal control, and treatment timing should consequently be matched to the patient’s soft-tissue and bone morphology [113].

Root morphology and the planned path of movement also contribute to risk. Orthodontically induced inflammatory root resorption (OIIRR) is more frequent or severe with heavy continuous forces, intrusion, torque, prolonged treatment, extraction therapy, and extensive apical displacement, particularly in maxillary incisors [2]. Evidence for many patient-related predictors remains inconsistent; therefore, risk assessment should combine baseline imaging and clinical history with periodic radiographic monitoring rather than rely on a single biomarker. When resorption is detected, reduction or temporary interruption of active force and modification of the treatment objective may be appropriate [2].

Genetic susceptibility to external apical root resorption has also been investigated, although the evidence remains preliminary. A systematic review reported associations for several candidate genes, but meta-analysis identified only P2RX7 rs208294, and the certainty of evidence was very low. Genetic testing is therefore not currently justified for routine root-resorption risk assessment; its future role, if confirmed, would more plausibly be complementary to mechanical and radiographic evaluation than a replacement for them [96].

#### 4.2.5. Genetic Background and the Limits of Prediction

Malocclusion, craniofacial growth, eruption disturbances, tissue remodeling, and susceptibility to adverse effects are complex traits produced by multiple genes interacting with environmental exposures [5]. A recent synthesis of skeletal malocclusion genetics identified replicated or biologically plausible signals across the GHR/IGF1, FGFR, WNT/β-catenin, TGF-β/BMP, RUNX2, MSX1, and GLI2 pathways, while also highlighting ancestry-specific effects and limited replication. In this context, proposed polygenic scores are better regarded as research tools than as diagnostic instruments at the present stage [51].

Candidate-gene studies illustrate both the potential and the limitations of this field. Variants in EGF and EGFR have been associated with mandibular retrognathism in a German adolescent sample [27]. The MMP-1 rs1799750 2G/2G genotype increased in frequency with open-bite severity in one small case–control study, although overall genotype and allele distributions were not significantly different [28]. These observations require replication in larger and ethnically diverse cohorts before clinical testing can be recommended.

Noncoding variation may also contribute to phenotypic heterogeneity. A proof-of-concept case report described multiple craniofacial and dental anomalies in a child carrying variants in MIR146A and MIR182, supporting further investigation of microRNA-mediated regulation but not establishing causality [26]. Likewise, association-rule analysis of MSX1, PAX9, and AXIN2 variants identified potentially informative genotype combinations for dental impaction in patients with hypodontia, despite a non-significant conventional regression result [25]. Such exploratory findings show why predictive models must be externally validated and compared with conventional clinical assessment.

**Figure 3:**
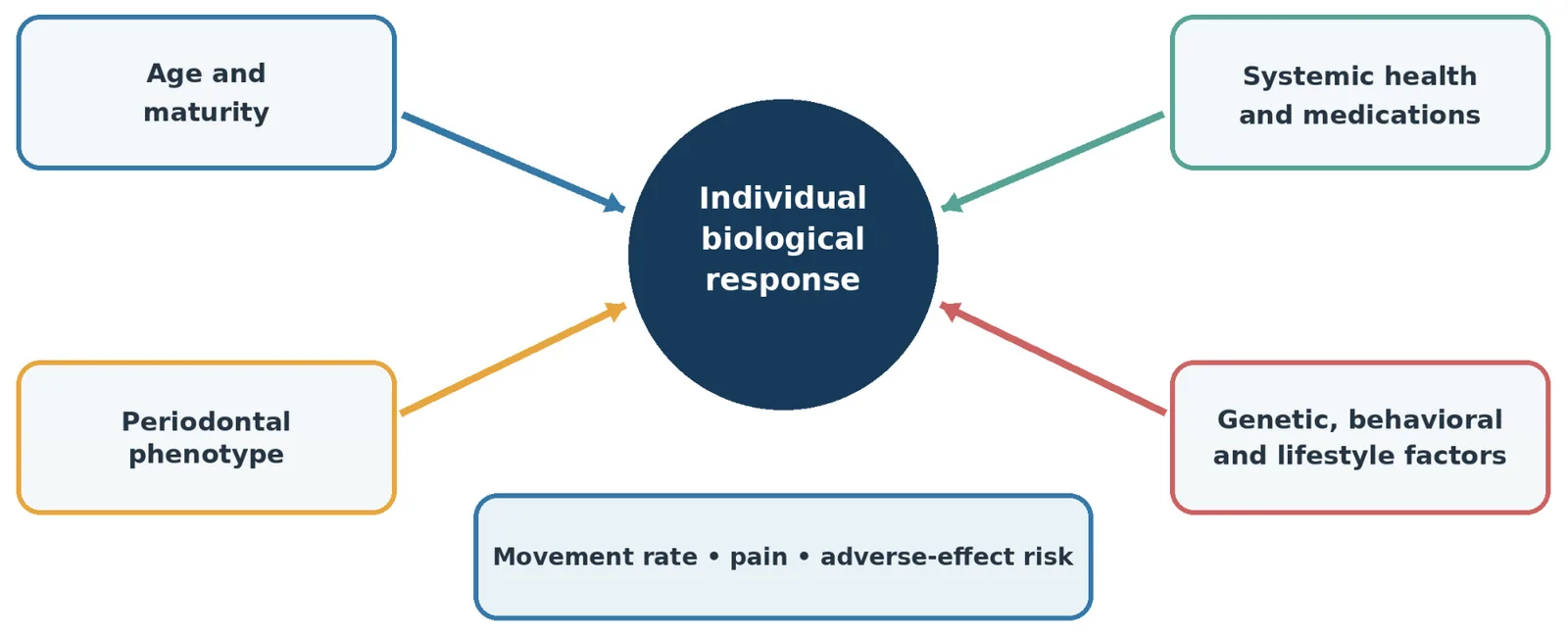
Principal biological, anatomical, systemic, behavioral, and genetic determinants of interindividual orthodontic response.

The principal determinants and clinical expressions of interindividual variability are summarized in Figure 3.

Genomic information should not be interpreted as a deterministic forecast. Treatment response emerges from interactions among genomic, epigenomic, transcriptomic, proteomic, metabolic, clinical, behavioral, and environmental variables. Personalized orthodontics is therefore more likely to depend on validated multivariable models than on isolated polymorphisms. Characterizing this variability through conventional clinical factors alone remains incomplete, thereby providing the rationale for investigating biomarkers, genomics, epigenetics, and other molecular approaches to patient phenotyping.

### 4.3. Emerging Biomedical Sciences in Orthodontics

#### 4.3.1. From Clinical Observation to Molecular Phenotyping

Conventional orthodontic monitoring is based mainly on clinical examination, imaging, appliance performance, and the observed amount of tooth movement. These measures remain indispensable, but they describe the result of remodeling rather than the biological processes that precede it. Biomarkers and omics technologies aim to characterize inflammation, extracellular matrix turnover, osteoclastogenesis, osteogenesis, cellular stress, and tissue damage while treatment is in progress. In principle, this information could distinguish normal adaptive remodeling from a response associated with delayed movement, periodontal damage, pain, or root resorption [3,6].

A clinically useful biomarker must be reproducible, temporally coherent, informative beyond routine variables, and linked to an actionable decision. Because the same molecule may reflect physiological remodeling or pathological inflammation depending on timing and concentration, longitudinal profiles should be interpreted alongside force characteristics, periodontal status, oral hygiene, age, medications, and movement type [6,108]. Although the included studies identified candidate molecular markers and treatment-related profiles, they did not establish the reproducibility of patient-level profiles or validate their ability to predict orthodontic treatment response in independent populations.

#### 4.3.2. Gingival Crevicular Fluid and Saliva as Diagnostic Media

GCF is closely associated with the periodontal tissues in which orthodontic remodeling occurs. Its minimally invasive collection permits repeated assessment of locally released cytokines, chemokines, enzymes, bone-turnover mediators, proteins, lipids, and nucleic acids. Saliva is easier to obtain and may be more suitable for large-scale or repeated screening, although dilution, flow rate, circadian variation, diet, oral hygiene, and contributions from multiple oral tissues can reduce site specificity. Standardization of sampling site, collection time, stimulation, storage, normalization, and analytical platform is therefore essential [3,77].

The most frequently studied inflammatory mediators include IL-1β, IL-6, TNF-α, prostaglandin E2, and chemokines involved in leukocyte recruitment. Their early increase after force application is consistent with the sterile inflammatory phase of OTM. MMPs, particularly MMP-8 and MMP-9, reflect extracellular matrix degradation, whereas alkaline phosphatase and osteocalcin are associated with bone formation. The RANKL/OPG ratio provides a biologically plausible indicator of the balance between osteoclastogenic stimulation and inhibition [3]. Longitudinal clinical data have reported early cytokine elevation, later increases in MMPs, and an increased RANKL/OPG ratio during active remodeling, supporting their potential as response markers [100]. Beyond inflammatory cytokines, longitudinal salivary proteomics has identified ApoE as a candidate marker of orthodontic remodeling. In 30 patients evaluated during early and later treatment phases, mass-spectrometric screening followed by Western blotting demonstrated stage-related differences in salivary ApoE; increased expression after force application was also confirmed immunohistochemically in a murine tooth-movement model. These findings support ApoE as a potential non-invasive indicator of alveolar remodeling, although its predictive value and specificity require validation in larger controlled cohorts [24].

#### 4.3.3. Proteomics, Spectroscopy, Metabolomics, and the Oral Microbiome

Proteomic analysis expands assessment beyond a small set of preselected cytokines. Integrated analysis of human GCF has identified temporally ordered protein networks related to inflammation, immune activity, bone resorption, extracellular matrix organization, and repair, reinforcing the concept that OTM progresses through overlapping biological phases [6]. Proteomic and phosphoproteomic approaches may also reveal signaling signatures that differ according to anatomical site. Human periodontal ligament stem cells from the maxilla and mandible have shown distinct kinase-activity responses to compression, suggesting a jaw-specific mechanobiological fingerprint that may contribute to heterogeneous remodeling [91].

Spectroscopic methods may provide rapid composite molecular information. Fourier-transform infrared spectroscopy of GCF can detect changes in protein, lipid, and carbohydrate bands, while attenuated-total-reflection sampling may shorten acquisition and preparation. Ratiometric spectral markers have therefore been proposed for monitoring force-induced tissue changes [17].

Metabolomics provides a downstream representation of cellular activity and host-microbial interaction. Salivary profiling after multibracket treatment has demonstrated substantial changes in metabolic pathways, indicating that orthodontic appliances alter the oral biochemical environment over time [23]. Combined microbiome-metabolome analysis has further shown that appliance type and baseline oral health influence microbial composition and metabolites, with some metabolite-microbe combinations emerging as candidate indicators of poor oral health [22]. These findings complement evidence that appliances can promote plaque retention and dysbiosis [118], but they do not yet establish biomarkers capable of directing routine appliance selection.

Cross-scale studies also illustrate how omics can link mechanics with cellular metabolism. In a clear-aligner model, regions of increased strain energy density showed greater osteoclastic activity, increased IL-1β, reduced BMP2, differential protein expression, and impaired mitochondrial oxidative phosphorylation. Although based partly on an animal model, this approach demonstrates how biomechanical mapping, proteomics, histology, and bioenergetics may be integrated into a biologically informed predictive system [119]. Personalized physical stimulation offers a related translational example. Peng et al. integrated human CT assessment with age-stratified animal experiments and reported that the frequency of vibration producing the greatest tooth-movement acceleration varied with jawbone density; combining vibration with near-infrared light further enhanced the response [117]. The study illustrates how an imaging-derived biological characteristic could inform selection of an adjunctive stimulus, while also emphasizing the need for prospective clinical validation before routine application.

#### 4.3.4. Genomics and Polygenic Architecture

Genomics investigates inherited variation contributing to craniofacial growth, malocclusion, eruption disturbances, remodeling, treatment response, and adverse effects. Relevant pathways include GHR/IGF1, FGF/FGFR, WNT/β-catenin, TGF-β/BMP, RUNX2, MSX1, PAX9, AXIN2, and GLI2. Because these phenotypes are polygenic and modified by ancestry and environment, current polygenic scores remain research tools rather than clinical tests [51].

Recent genetic studies illustrate both the appeal and the current limits of risk prediction. EGF and EGFR variants have been associated with mandibular retrognathism in a specific adolescent population [27], while an MMP-1 variant showed a relationship with open-bite severity despite non-significant overall genotype differences [28]. Exploratory combinations of MSX1, PAX9, and AXIN2 genotypes have also been evaluated for dental impaction in hypodontia [25]. Such associations may contribute to future multivariable risk models, but they should not be read as deterministic predictors in individual patients. Clinical genetic testing would need to improve diagnosis, prognosis, or treatment selection beyond conventional assessment before it could be justified.

The same caution applies to susceptibility to orthodontically induced external apical root resorption. Candidate-gene research has produced numerous associations, yet meta-analytic evidence remains sparse and of very low certainty [96]. Genomics may ultimately contribute one layer to a multivariable risk model combining root morphology, previous trauma, planned apical displacement, force exposure, treatment duration, and serial imaging, but it cannot currently replace these established factors.

#### 4.3.5. Noncoding RNAs and Post-Transcriptional Regulation

NcRNAs regulate gene expression without encoding proteins and include miRNAs, lncRNAs, and circRNAs. During OTM, these molecules can influence inflammatory signaling, osteoclast differentiation, osteoblastogenesis, periodontal ligament remodeling, angiogenesis, and extracellular matrix turnover [7]. Because miRNAs are relatively stable in saliva and GCF, they are attractive candidates for non-invasive monitoring. A systematic review identified orthodontically relevant changes in miR-21, miR-27, miR-29, miR-34, miR-101, miR-146, and miR-214, together with predicted networks involving collagens, growth-factor signaling, and matrix remodeling [77].

Experimental studies show that miRNA expression differs between compressive and tensile environments and can regulate RANKL-related osteoclastogenesis, Wnt signaling, osteogenic differentiation, and inflammatory pathways [7,52]. This provides a plausible basis for diagnostic and therapeutic applications, but the evidence is still derived predominantly from preclinical models.

Clinical evidence has begun to test whether these mechanistic observations translate to measurable patient-level differences. In a randomized split-mouth trial involving 21 healthy orthodontic patients, Polizzi et al. evaluated serial GCF expression of miR-7, miR-21, miR-100, miR-125b, and miR-200b on the compression and tension sides during canine distalization. OTM produced side- and time-dependent miRNA dysregulation, while higher baseline miR-7a-2-3p, miR-21-5p, and miR-200b-3p, lower miR-125b-2-3p, younger age, and lower bleeding on probing were associated with greater movement at 3 months [19]. The study is important because it links molecular profiles with subsequent tooth movement in humans; however, its small sample and short follow-up mean that GCF miRNAs cannot yet be used as standalone clinical predictors.

Clinical translation is still preliminary. In a five-patient pilot study, miR-200a was detectable in saliva and serum before and after functional-appliance therapy, but changes were not statistically significant. The study supports feasibility of non-invasive detection rather than validation of miR-200a as a condylar-growth biomarker [15]. Similarly, a proof-of-concept case report associating MIR146A and MIR182 variants with craniofacial and dental anomalies generates a biologically plausible hypothesis but cannot establish causality or predictive performance [26].

#### 4.3.6. Epigenetics and Mechanical Memory

Epigenetic mechanisms—including DNA methylation, histone modification, chromatin accessibility, and ncRNA networks—respond to mechanical stress, inflammation, oxygen tension, metabolism, age, and disease. Persistent cell-state changes after loading have been described as mechanical memory and may contribute to variation in adaptation or relapse, although their duration in human orthodontic tissues remains uncertain [8].

Dental-derived mesenchymal stem-cell populations possess distinct methylation landscapes related to odontogenesis, osteogenesis, neural development, and tissue-specific differentiation. Integrated analysis has identified regulatory networks involving PAX6, FOXC2, NR2F2, SALL1, BMP7, JAG1, and multiple ncRNAs, suggesting candidate biomarkers and regenerative targets [59]. These profiles are primarily mechanistic and should not yet be interpreted as chairside diagnostic signatures.

Histone modifications also connect metabolism with mechanosensitive inflammation. In force-stressed PDL fibroblasts, oleic-acid-related anti-inflammatory effects were associated with altered histone H3 lysine acetylation and IL10 expression [103]. Together with mechanically induced methylation and chromatin changes [8], this supports convergence among genetic background, metabolism, and loading at the epigenetic level. The resulting volume and complexity of molecular information exceed what can be interpreted reliably through isolated variables, creating a natural role for computational methods capable of integrating biological, imaging, biomechanical, and clinical data.

**Figure 4:**
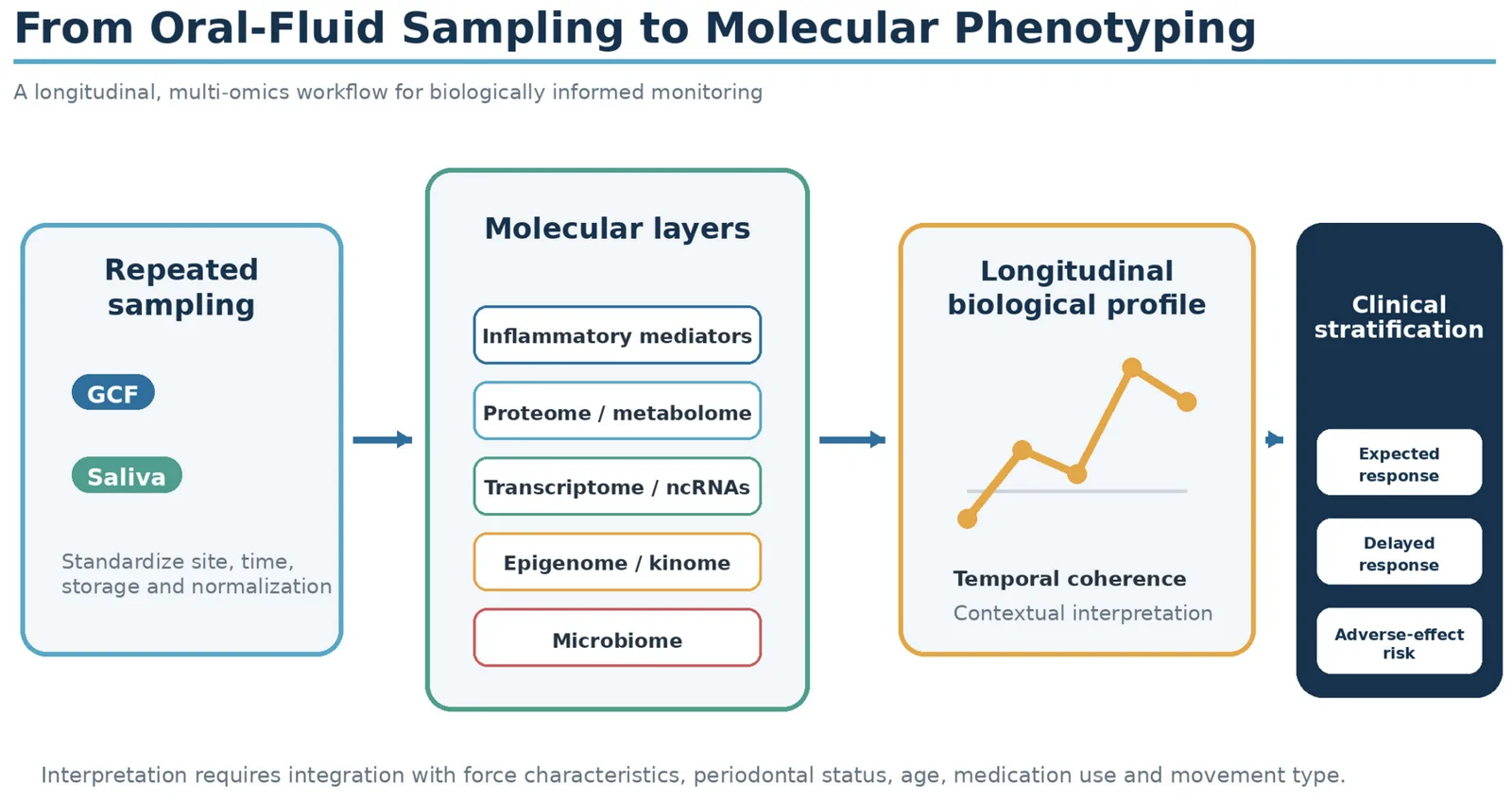
Proposed conceptual workflow for integrating repeated oral-fluid sampling, biomarkers, and multi-omics data into longitudinal biological phenotyping and clinical stratification. The workflow reflects the authors’ synthesis of the included studies and contextual literature and has not been clinically validated.

Figure 4 presents a proposed pathway for longitudinal molecular phenotyping, developed from the authors’ synthesis of the included studies and contextual literature; it has not been validated as a clinical pathway.

### 4.4. The Role of Artificial Intelligence in Personalized Orthodontics

#### 4.4.1. Artificial Intelligence as a Clinical Support System

The systematically included evidence on computational methods was limited to genetic association-rule analysis, gingival-enlargement classification, and root-resorption measurement [18,20,25]. The other AI applications discussed in this section are drawn from contextual literature or represent proposed directions; they were not evaluated across the 14 included studies.

The three included computational applications addressed distinct tasks. Alhazmi et al. [25] analyzed genetic association rules in 106 participants; the reported lift does not constitute validated individual-level predictive performance. Navarro-Fraile et al. [18] used AI-assisted segmentation and measurement to quantify root resorption in a preliminary randomized study of 18 patients; AI did not determine force selection. Simancas-Escorcia et al. [20] reported discrimination for gingival enlargement using S100A4-positive fibroblast density in 152 tissue samples, but the reported AUC was not supported by prospective external validation. These studies cannot be combined into a single estimate of AI performance. In particular, the available reports do not establish generalizable performance across independent clinical settings or improved outcomes from AI-supported decisions.

AI includes computational methods capable of recognizing patterns, classifying data, estimating probabilities, and generating predictions. ML derives relationships from structured variables, whereas deep learning (DL) is particularly suited to radiographs, photographs, surface scans, and other high-dimensional inputs. In orthodontics, these methods support landmark detection, skeletal-age assessment, pathology screening, treatment planning, outcome prediction, and remote monitoring [4].

AI-supported personalization seeks to integrate facial and skeletal phenotype, dental position, periodontal anatomy, growth, biomechanics, systemic history, behavior, and—when validated—molecular information [71,73,76]. Its output must be interpreted within the population, data quality, reference standard, and clinical context for which the model was developed [4].

#### 4.4.2. Imaging, Segmentation, and Automated Diagnosis

Automated cephalometric landmark identification is among the most mature orthodontic applications. Related approaches support three-dimensional landmarking, tooth and root segmentation, airway analysis, cervical vertebral maturation, and skeletal-pattern classification [4,18]. AI-assisted analysis of cone-beam computed tomography (CBCT), intraoral and facial scans, may improve visualization of root position, alveolar boundaries, impacted teeth, temporomandibular structures, and surgical relationships [4,73]. Nevertheless, artifacts, voxel size, metallic appliances, atypical morphology, and inconsistent labeling may reduce robustness, making quality-control procedures and clinician correction essential.

Root-resorption assessment illustrates both the promise and task-specific limitations of automated imaging. A preliminary randomized trial used AI-aided segmentation to quantify three-dimensional changes in root volume and length, but disagreement with manual measurements remained [2,18]. An error acceptable for screening may therefore be unsuitable for evaluating individual roots, alveolar limits, asymmetric anatomy, or surgical movements.

#### 4.4.3. Treatment Planning, Simulation, and Outcome Prediction

AI can contribute to extraction versus non-extraction assessment, estimates of treatment complexity or duration, aligner staging, orthognathic planning, and the identification of patients who may need closer surveillance [4]. For these outputs to be clinically useful, systems should report calibrated probabilities and uncertainty and make clear which variables drive an estimate, allowing the result to be judged against biomechanical feasibility and patient preferences. Digital setups illustrate the distinction between computational planning and biological prediction: they depict intended tooth positions but may not adequately represent periodontal phenotype, root morphology, force decay, anchorage, adherence, or individual remodeling capacity [71,73,113].

Predicting adverse effects is a potential application, but the included evidence has not established prospective predictive performance. In a cross-sectional study, S100A4-positive fibroblast density discriminated between groups with and without orthodontically induced gingival enlargement within the study sample [20]. Similar approaches are being explored for root resorption, periodontal deterioration, pain, relapse, and treatment discontinuation [2,96,112], although evidence for clinical deployment remains limited.

#### 4.4.4. Integrating AI with Biomarkers, Genomics, and Omics

The main biomedical opportunity for AI lies in integrating complementary data types. Biomarkers are time-dependent and influenced by periodontal status and sampling conditions; genomic variants generally have small, population-dependent effects; imaging defines anatomical boundaries; and biomechanical models estimate tissue loading. ML methods may identify nonlinear interactions among these domains that are difficult to detect using conventional analyses [6,17,18,22,23,51,59,77,91,100,103,119].

Early studies have applied association-rule analysis to MSX1, PAX9, and AXIN2 variants [25], linked microbial and metabolomic patterns with oral-health indicators [22], and combined biomechanical and molecular data to characterize periodontal remodeling [119]. These applications illustrate the potential of nonlinear integration but remain exploratory. Longitudinal measurements may be particularly valuable because inflammatory, metabolic, and remodeling signals vary across treatment phases.

#### 4.4.5. Remote Monitoring, Smart Appliances, and Adaptive Treatment

Remote-monitoring platforms use patient-generated photographs, videos, or scans to assess aligner fit, bracket integrity, oral hygiene, and approximate tooth movement. Automated triage can facilitate earlier review and may reduce unnecessary appointments, but variable image quality, patient technique, unequal access to technology, false reassurance, and missed pathology limit what can safely be delegated to remote assessment [4,18]. For this reason, these tools are best used as an adjunct to, rather than a substitute for, scheduled clinical and radiographic examinations.

Sensor-equipped brackets or aligners could collect force, temperature, wear-time, or movement data and transmit them through connected systems. AI could then compare the observed response with the planned trajectory and prompt clinical review. These concepts remain largely experimental and require assessment of accuracy, biocompatibility, cybersecurity, durability, cost, and clinically relevant safety outcomes [1,2,43,108,112,113].

#### 4.4.6. Validation, Governance, Adoption, and the Human–AI Relationship

Clinical implementation requires external and prospective validation using appropriate comparators and patient-relevant outcomes. Reports should include calibration, uncertainty, subgroup performance, failure cases, and clinician interaction, with post-deployment monitoring for dataset shift [4,18]. Bias arising from underrepresentation or inconsistent labels must be addressed, while craniofacial and genomic data require informed consent, minimization, security, auditability, and defined responsibility [51,66]. Explainability should be proportional to the clinical risk of the supported decision.

Adoption also depends on professional competence and trust. Although attitudes toward AI are generally favorable, knowledge and routine use remain uneven, with concerns regarding cost, privacy, legal responsibility, patient interaction, and clinical errors [84]. Training should address data quality, performance, bias, uncertainty, and recognition of implausible output; generative AI should not be treated as autonomous diagnostic evidence [4,84].

A clinically realistic model is therefore one of shared roles rather than autonomous decision-making. Computational tools can organize and quantify complex information, while biological knowledge, biomechanics, patient preferences, and professional judgment remain necessary to interpret that information and determine the final decision [4,76,84,103]. Moving from this form of decision support to routine personalized care will depend less on adding further algorithms than on resolving persistent problems of validation, generalizability, governance, and implementation.

The included computational studies did not collectively provide sufficient evidence on patient-independent validation, external testing, calibration, class imbalance, missing-data handling, or protection against overfitting to establish clinical transportability. These reporting and validation gaps should be addressed explicitly in future studies.

**Figure 5:**
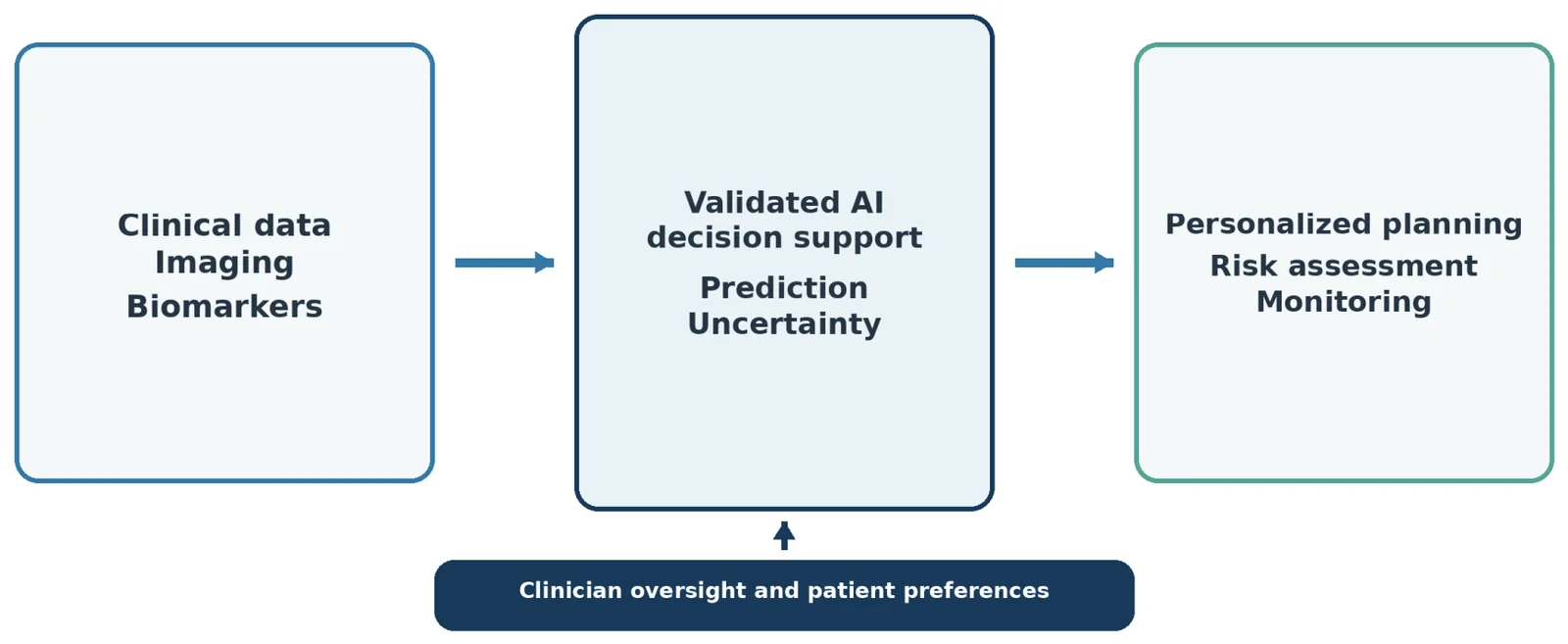
Proposed conceptual framework for integrating clinical, imaging, biomechanical, molecular, and longitudinal data with AI under clinician oversight. This framework was derived from the authors’ synthesis and contextual literature and has not been clinically validated by the included studies.

The proposed multimodal framework for AI-supported personalized orthodontics is illustrated in Figure 5.

### 4.5. Limitations and Future Research Directions

#### 4.5.1. Limitations

The principal limitation is the restricted and heterogeneous human evidence base. Given the breadth of the review question and the small number of eligible studies within each outcome area, the available evidence may be incomplete. Publication bias and selective reporting remain possible, but the sparse and heterogeneous data did not permit a reliable formal assessment. Several included studies were exploratory, single-center investigations with small samples, limited follow-up, incomplete control of confounding, or no external validation. The diversity of populations, interventions, biological specimens, sampling schedules, analytical platforms, and outcomes prevented quantitative pooling and limits direct comparison. Risk of bias was evaluated using design-appropriate instruments for all 14 included human studies; nevertheless, these judgments do not resolve concerns regarding imprecision, applicability, or generalizability. The outcome-level certainty assessment was predominantly very low for proposed clinical personalization applications. Several conclusions were supported by a single small study, and certainty regarding AI-guided force selection and genetic prediction of treatment response could not be rated because direct studies were unavailable. Reviews, book chapters, animal models, in vitro studies, finite element analyses, and conceptual reports were used solely for contextual interpretation and were not subjected to the clinical risk-of-bias assessment. Consequently, mechanistic plausibility from these sources should not be interpreted as equivalent to human clinical effectiveness or as supporting graded treatment recommendations.

Requiring personalization terminology in the database searches may have reduced retrieval of relevant studies that did not use these terms in their titles or abstracts. The findings should therefore be interpreted within the defined scope of the search rather than as an exhaustive synthesis of all primary studies on orthodontic biological responses and emerging molecular methods.

In addition, the full texts of six potentially relevant reports could not be retrieved, and eligibility was restricted to English-language publications. These review-process limitations may have resulted in the omission of relevant evidence and further limit the completeness and generalizability of the synthesis.

Finally, Embase was not available through our institutional access, and IEEE Xplore was not searched because the review focused on biomedical and clinical applications of personalized orthodontics. Although PubMed, Scopus, and Web of Science cover the relevant biomedical and computational literature, searching these additional databases might have identified further eligible studies, particularly reports indexed only in those sources.

#### 4.5.2. Future Research Directions

The next stage of research needs to test personalization as a clinical strategy rather than only as a collection of promising markers or models. Multicenter longitudinal cohorts could combine standardized phenotyping with quantified biomechanics, three-dimensional imaging, periodontal and root outcomes, patient-reported measures, and repeated biological sampling [15,17,22,23,52,59,71,73,76,77,91,100,103,108,113,119]. Future studies should prespecify patient-relevant outcomes, recruit sufficiently powered multicenter cohorts, standardize phenotyping and biomarker assays, and use independent validation cohorts. AI studies should also report their data sources, model-development and validation procedures, performance, and handling of missing data and overfitting transparently. For biomarkers and AI systems, the relevant question is whether they add useful information to conventional assessment while remaining feasible, equitable, and safe [22,25,51,59,66,76]. Ultimately, randomized or pragmatic studies will be needed to establish whether biologically or computationally guided treatment improves patient-centred outcomes without increasing periodontal or root-related harm [18,20,96,100,112,113].

## 5. Conclusions

The included human studies documented changes in inflammatory mediators, gene expression, and miRNAs during OTM, but did not establish clinically validated thresholds for predicting an individual patient’s response. Studies of biological variability examined force-related outcomes and pretreatment dental or craniofacial phenotypes; the latter did not test whether genetic variants predict treatment response. Biomarker, spectroscopy, metabolomic, and microbiome studies identified candidate measures of treatment-related change or oral health, without externally validated clinical prediction. The included computational studies assessed genetic association rules, gingival-enlargement classification, and AI-assisted root-resorption measurement; none demonstrated improved outcomes from AI-guided force selection.

Certainty in the evidence supporting clinical personalization applications was predominantly very low, owing to small samples, methodological limitations, imprecision, and indirectness to treatment decisions. No certainty rating could be assigned to AI-guided force selection or genetic prediction of treatment response because the included studies did not directly evaluate those claims. At present, treatment decisions should remain grounded in established clinical and biomechanical assessment; the additional clinical value of molecular and computational measures requires prospective validation.

## Figures and Tables

**Table 1:** Search terms used for each database. The asterisk (*) indicates the truncation wildcard used to retrieve multiple word variants sharing the same root.

Database	Search Format
PUBMED	((orthodontic*[Title/Abstract] OR orthodontics[MeSH Terms] OR orthodontics[Title/Abstract]) AND (personalized[Title/Abstract] OR personalised[Title/Abstract] OR precision[Title/Abstract] OR individualized[Title/Abstract] OR individualised[Title/Abstract])) AND (genomic*[Title/Abstract] OR genetic*[Title/Abstract] OR biolog*[Title/Abstract] OR biomarker*[Title/Abstract] OR epigenetic*[Title/Abstract] OR “artificial intelligence”[Title/Abstract] OR AI[Title/Abstract])
WEB OF SCIENCE (All databases)	TS = ((orthodontic* OR orthodontics) AND (personalized OR personalised OR precision OR individualized OR individualised)) AND TS = (genomic* OR genetic* OR biolog* OR biomarker* OR epigenetic* OR “artificial intelligence” OR AI)
SCOPUS	(TITLE-ABS-KEY ((orthodontic* OR orthodontics) AND (personalized OR personalised OR precision OR individualized OR individualised)) AND TITLE-ABS-KEY (genomic* OR genetic* OR biolog* OR biomarker* OR epigenetic* OR “artificial intelligence” OR AI))

**Table 2:** Characteristics of the primary human studies included in the systematic review. Study-level risk-of-bias assessments are reported in Supplementary Table S2; outcome-level certainty assessments are reported in Supplementary Table S3.

Author, Year, and Location	Sample	Study Design	Objective	Results	Conclusion	Relationship to Personalized Care
Alhazmi et al. [25], 2026, Saudi Arabia	106 participants: 54 patients with hypodontia and 52 controls	Case–control genetic association study with association-rule analysis	Evaluate combinations of candidate genetic variants associated with hypodontia and dental impaction and explore their predictive patterns using association-rule algorithms.	Among 106 participants, logistic regression did not identify a significant association between the investigated genotypes and dental impaction (*p* = 0.112). Association-rule analysis yielded the highest lift for PAX9 C/C combined with MSX1 A/A (lift = 1.671); external predictive performance was not reported	Specific variant combinations showed potentially informative associations, but broad age distribution, limited confounder adjustment, and absence of external validation preclude clinical predictive use.	Enabling patient-stratification evidence
Grenga et al. [26], 2025, Italy	1 patient carrying two microRNA variants	Case report	Explore whether rare miRNA variants could help explain a complex craniofacial and dental phenotype.	One patient with impacted canines, odontoma, root resorption, and skeletal anomalies carried MIR146A rs2910164 and MIR182 rs76481776 variants. No comparison group or association estimate was available.	Rare regulatory variants may contribute to craniofacial abnormalities, but causality cannot be inferred.	Enabling patient-stratification evidence
Kapoor et al. [15], 2025, India	5 children aged 9–11, before and 9 months after therapy	Pilot longitudinal clinical study	Assess salivary and serum miR-200a during functional-appliance–related condylar adaptation.	In five children, salivary and serum miRNA-200a ΔCt did not change significantly nine months after functional-appliance therapy (*p* = 0.686 and *p* = 0.715, respectively). One post-treatment serum sample could not be analyzed.	The marker is biologically plausible but requires adequately powered validation.	Direct personalization evidence
Kumar et al. [16], 2022, India	20 patients, 11–28 years	Prospective clinical study	Compare early GCF cytokine responses between adolescents and young adults.	In 20 patients, GCF IL-1 increased from 15.67 to 27.94 pg/L after force activation (*p* = 0.009). The post-activation value was higher in adolescents than in young adults (42.96 versus 17.93 pg/L; *p* = 0.006).	Age-related inflammatory differences may partly explain variation in early tooth movement.	Direct personalization evidence
Lepore et al. [17], 2025, Italy	6 patients, GCF at baseline, 2 days and 2 weeks	Prospective pilot spectroscopy study	Test a faster Attenuated total reflection (ATR)—Fourier transform infrared (FTIR) workflow for monitoring biochemical GCF changes during OTM.	GCF spectra from six patients differed between baseline, two days, and two weeks after treatment initiation. The 1237/1651 cm^−1^ band-area ratio changed significantly (*p* < 0.05); prediction of a clinical outcome was not evaluated.	Rapid spectroscopy may facilitate monitoring after larger clinical validation.	Direct personalization evidence
Navarro-Fraile et al. [18], 2025, Spain	18 randomized patients from a planned cohort of 43	Preliminary two-arm randomized clinical trial	Compare individualized versus conventional force sequences and manual versus AI resorption measurement.	In 18 randomized patients, AI-measured root-length loss was 0.20 ± 0.23 mm with conventional forces versus 0.42 ± 0.43 mm with individualized forces after 142 days (*p* = 0.045). Total root-volume loss was 2.44 ± 6.59 versus 2.42 ± 4.75 mm^3^ (*p* > 0.05). AI measured resorption; it did not select the forces.	AI can accelerate quantification, but measurement agreement and clinical thresholds need refinement.	Direct personalization evidence
Paddenberg-Schubert et al. [27], 2025, Germany	119 patients aged 10–18: 74 retrognathic, 45 controls	Case–control study	Test associations of epidermal growth factor (EGF)/epidermal growth factor receptor (EGFR) polymorphisms with mandibular retrognathism.	Among 45 orthognathic controls and 74 retrognathic cases, the EGF rs4444903 minor allele and the EGF rs4444903|rs2237051 haplotype were more frequent in controls (*p* = 0.008 and *p* = 0.007, respectively). Treatment response was not assessed.	These variants are promising candidates but require replication before screening use.	Enabling patient-stratification evidence
Polizzi et al. [19], 2024, Italy	21 healthy orthodontic patients (mean age 13.2 ± 1.8 years); split-mouth canine distalization model	Randomized controlled split-mouth clinical trial	Evaluate compression- and tension-side GCF miRNA profiles during OTM and identify baseline molecular and clinical predictors of movement over 3 months.	In 21 patients, GCF microRNA expression changed on compression and tension sides over three months. Baseline miRNA-7a-2-3p, miRNA-21-5p, miRNA-200b-3p, miRNA-125b-2-3p, age, and bleeding on probing were associated with three-month tooth movement in the reported multivariable model (*p* = 0.014–0.042); externally validated prediction was not reported.	OTM produced side- and time-dependent miRNA dysregulation. Baseline miRNA profiles, younger age, and lower gingival bleeding were associated with 3-month movement, supporting GCF miRNAs as candidate biomarkers that require larger and longer validation.	Direct personalization evidence
Simancas-Escorcia et al. [20], 2026, Colombia/India	152 tissue samples: 60 healthy, 31 orthodontic controls, 61 Orthodontic-induced gingival enlargement (OIGE)	Cross-sectional study	Evaluate S100A4-positive fibroblasts for early OIGE risk stratification.	S100A4-positive fibroblast density was 245.8 ± 38.7 cells/mm^2^ in gingival-enlargement samples, 165.3 ± 29.4 in orthodontic controls, and 98.2 ± 18.5 in healthy controls (*p* < 0.001). The reported adjusted OR was 1.028 per cell/mm^2^ (95% CI 1.021–1.035), with AUC = 0.952 (95% CI 0.921–0.983); prospective external validation was not reported.	S100A4 is a promising risk marker, but prospective external validation is required before screening.	Direct personalization evidence
Spitz et al. [21], 2022, Brazil	15 participants: movement alone vs movement plus micro-osteoperforations (MOPs)	Randomized clinical study	Determine whether MOPs alter the PDL transcriptome during human tooth movement.	Seven participants received tooth movement alone and eight received tooth movement plus micro-osteoperforations. Compared with unloaded contralateral teeth, 331 and 356 genes, respectively, were differentially expressed after seven days. No genes met the prespecified differential-expression threshold in the direct comparison between treatment groups.	MOPs induce a measurable molecular response, but clinical benefit requires larger outcome-based trials.	Direct personalization evidence
Velachery et al. [23], 2025, India	6 patients before and two weeks after debonding	Longitudinal clinical study	Characterize salivary metabolic changes associated with fixed-appliance removal.	In paired saliva samples from six patients, 2153 metabolites were identified; 320 occurred at both time points, and 50 shared metabolites showed a significant fold change two weeks after debonding. No untreated comparison group was studied.	Salivary metabolomics can detect treatment-related change but needs larger controlled studies.	Direct personalization evidence
Xie et al. [22], 2025, China	61 adolescents in six appliance/oral-hygiene groups	Cross-sectional study	Compare microbiome, metabolome and health indicators across aligner, fixed-appliance and control groups.	In 61 adolescents, oral health status was more strongly associated with microbiota variation than appliance type (ANOSIM *R* = 0.29 versus 0.09; both *p* = 0.001). Of 1977 detected metabolites, 13 were selected as candidate markers associated with poor oral health; external predictive validation was not reported.	Multi-omics may support individualized prevention, but longitudinal confirmation is necessary.	Direct personalization evidence
Yılmaz et al. [28], 2025, Türkiye	30 open-bite patients and 30 controls, aged 15–35	Case–control study	Assess whether MMP-1 rs1799750 relates to open bite or its severity.	Genotype and allele distributions for MMP-1 rs1799750 did not differ significantly between 30 open-bite cases and 30 controls (*p* = 0.2269 and *p* = 0.7125). Genotype distribution varied across open-bite severity categories (*p* = 0.009); the case group deviated from Hardy–Weinberg equilibrium (*p* = 0.015).	The variant may influence severity, although the association is exploratory.	Enabling patient-stratification evidence
Zhang et al. [24], 2022, China	30 orthodontic patients assessed at early and 12-month phases; complementary mouse OTM model	Longitudinal clinical study with animal validation	Investigate whether salivary apolipoprotein E changes during treatment and may serve as a marker of orthodontic remodeling.	In 30 patients assessed at approximately two and 12 months, salivary peptide profiling and Western blotting identified a treatment-stage difference in ApoE expression. An effect magnitude, patient-level prediction threshold, and discrimination statistic for treatment response were not established.	Salivary apolipoprotein E (ApoE) is a plausible non-invasive marker of force-related alveolar remodeling, but predictive performance requires larger controlled validation.	Direct personalization evidence

**Table 3:** Methodological characteristics and validation of computational approaches in the 14 included studies. (A) Methods and dataset characteristics. (B) Performance and clinical validation.

(A)					
Study	AI Methodology and Task	Dataset Size	Dataset Source and Characteristics	Training, Validation, and Test Strategy	Patient-Independent Data
Alhazmi et al. [25]	Apriori association-rule analysis of genetic variants and dental impaction; logistic regression	106 participants (54 with hypodontia; 52 controls)	Two orthodontic clinics in Riyadh; clinical/radiographic assessment and salivary genotypes	Train/test splitting and internal k-fold cross-validation described; split proportions and held-out test results NR	Patient-level records; independence of training/test partitions insufficiently described
Grenga et al. [26]	Not applicable	Not applicable	Not applicable	Not applicable	Not applicable
Kapoor et al. [15]	Not applicable	Not applicable	Not applicable	Not applicable	Not applicable
Kumar et al. [16]	Not applicable	Not applicable	Not applicable	Not applicable	Not applicable
Lepore et al. [17]	Not applicable	Not applicable	Not applicable	Not applicable	Not applicable
Navarro-Fraile et al. [18]	Deep-learning-assisted CBCT tooth segmentation and root-resorption measurement; no AI-guided force selection	18 randomized orthodontic patients, from a planned sample of 43	Single-center clinical trial in Madrid; sectorial CBCT scans and maxillary-incisor segmentation	AI tool applied to trial images; original AI development dataset, training/test split, and model-validation strategy NR	Patient-level evaluation in trial; independence of the AI tool’s original development data NR
Paddenberg-Schubert et al. [27]	Not applicable	Not applicable	Not applicable	Not applicable	Not applicable
Polizzi et al. [19]	Not applicable	Not applicable	Not applicable	Not applicable	Not applicable
Simancas-Escorcia et al. [20]	Random Forest and support-vector-machine classification using S100A4-positive fibroblast density	152 tissue samples (60 healthy; 31 orthodontic controls; 61 gingival enlargement)	Clinical gingival tissue samples; cross-sectional analysis	Tenfold cross-validation and bootstrap (1000 iterations); separate external test cohort NR	Patient independence of validation partitions NR
Spitz et al. [21]	Not applicable	Not applicable	Not applicable	Not applicable	Not applicable
Velachery et al. [23]	Not applicable	Not applicable	Not applicable	Not applicable	Not applicable
Xie et al. [22]	Not applicable	Not applicable	Not applicable	Not applicable	Not applicable
Yılmaz et al. [28]	Not applicable	Not applicable	Not applicable	Not applicable	Not applicable
Zhang et al. [24]	Not applicable	Not applicable	Not applicable	Not applicable	Not applicable
**(B)**					
**Study**	**Performance Metrics**	**Internal Versus External Validation**	**Class Imbalance, Missing Data, and Overfitting**	**Comparator or Standard-of-Care Method**	**Prospective Evaluation**
Alhazmi et al. [25]	Highest rule lift 1.671; logistic-model accuracy 0.95, but precision, recall, and F1 score all 0	Internal k-fold cross-validation reported; no independent external validation	Missing genotypes: listwise deletion (<5%). Class-imbalance handling not reported (NR); cross-validation used to address overfitting	Logistic regression as analytical comparator; no prospective standard-of-care decision comparison	No; case–control analysis
Grenga et al. [26]	Not applicable	Not applicable	Not applicable	Not applicable	Not applicable
Kapoor et al. [15]	Not applicable	Not applicable	Not applicable	Not applicable	Not applicable
Kumar et al. [16]	Not applicable	Not applicable	Not applicable	Not applicable	Not applicable
Lepore et al. [17]	Not applicable	Not applicable	Not applicable	Not applicable	Not applicable
Navarro-Fraile et al. [18]	Root length and volume loss reported; no metric of AI prediction of treatment response	Comparison with manual segmentation in trial; no independent external validation of AI model reported here	Original AI training: class imbalance, missing-data handling, and overfitting procedures NR	Manual segmentation; conventional versus individualized archwires compared separately	Clinical outcomes prospectively assessed; AI not prospectively evaluated as decision support
Paddenberg-Schubert et al. [27]	Not applicable	Not applicable	Not applicable	Not applicable	Not applicable
Polizzi et al. [19]	Not applicable	Not applicable	Not applicable	Not applicable	Not applicable
Simancas-Escorcia et al. [20]	Primary receiver operating characteristic (ROC) area under the curve (AUC) 0.952; Random Forest AUC 0.952; support-vector-machine AUC 0.946	Internal resampling reported; no independent external validation	Specific missing-data and class-imbalance procedures NR; resampling was reported, but additional overfitting controls NR	Clinical variables and conventional regression analyses	No; cross-sectional study
Spitz et al. [21]	Not applicable	Not applicable	Not applicable	Not applicable	Not applicable
Velachery et al. [23]	Not applicable	Not applicable	Not applicable	Not applicable	Not applicable
Xie et al. [22]	Not applicable	Not applicable	Not applicable	Not applicable	Not applicable
Yılmaz et al. [28]	Not applicable	Not applicable	Not applicable	Not applicable	Not applicable
Zhang et al. [24]	Not applicable	Not applicable	Not applicable	Not applicable	Not applicable

## Data Availability

The original contributions presented in this study are included in the article/Supplementary Material. Further inquiries can be directed to the corresponding author.

## Supplementary Materials

The following supporting information can be downloaded at https://www.mdpi.com/article/10.3390/jcm15207767/s1: Table S1: Completed PRISMA 2020 checklist [10]; Table S2, Design-specific risk-of-bias assessment of clinical in vivo human studies included in the review [11,12,13]. Table S3: Outcome-level certainty-of-evidence assessments and supporting judgments [14,15,16,17,18,19,20,21,22,23,24,25,26,27,28]. Table S4: Reason for article exclusion after full-text assessment [1,2,3,4,5,6,7,9,29,30,31,32,33,34,35,36,37,38,39,40,41,42,43,44,45,46,47,48,49,50,51,52,53,54,55,56,57,58,59,60,61,62,63,64,65,66,67,68,69,70,71,72,73,74,75,76,77,78,79,80,81,82,83,84,85,86,87,88,89,90,91,92,93,94,95,96,97,98,99,100,101,102,103,104,105,106,107,108,109,110,111,112,113,114,115,116].

## Abbreviations

The following abbreviations are used in this manuscript:

PRISMA	Preferred Reporting Items for Systematic Reviews and Meta-Analyses
PROSPERO	Prospective Register of Systematic Reviews
SR	Systematic review
OTM	orthodontic tooth movement
PDL	Periodontal ligament
GCF	Gingival crevicular fluid
IL-1β	Interleukin-1β
TNF-α	Tumor necrosis factor-α
OPG	Osteoprotegerin
MMP	Matrix metalloproteinases
miRNAs	microRNAs
AI	Artificial intelligence
ML	Machine learning
ncRNAs	noncoding RNAs
lncRNAs	Long-noncoding RNAs
circRNAs	Circular RNAs
PDLSCs	Periodontal ligament stem cells
SED	strain energy density
FEM	finite element modeling
FTIR	Fourier transform infrared
ATR	Attenuated total reflection
EGF	Epidermal growth factor
EGFR	Epidermal growth factor receptor
CT	Computed tomography
EARR	External apical root resorption
OIGE	Orthodontic-induced gingival enlargement
MOPs	Micro-osteoperforations
ApoE	Apolipoprotein E
NSAIDs	Nonsteroidal anti-inflammatory drugs
OIIRR	Orthodontically induced inflammatory root resorption
DL	Deep Learning
CBCT	cone-beam computed tomography
