# Supplementary material for: Biological Variability and Biomedical Innovation in Personalized Orthodontics: A Systematic Review with Narrative Synthesis
Source: J Clin Med. 2026 Oct 9;15(20):7767. doi: 10.3390/jcm15207767 (PMC13705211; doi:10.3390/jcm15207767)
Supplement: Supplementary file 1 [file jcm-15-07767-s001.zip › Supplementary_Table_S2_full_reappraisal.pdf]

Table S2. Design-specific risk-of-bias assessment of clinical in vivo human studies included in the review [11–13].

A. Randomized trials: RoB 2

Target: effect of assignment to intervention on the principal reported clinical or molecular outcome. D1 randomization; D2 deviations; D3 missing outcome data; D4 measurement; D5 selection of reported result. L = low risk; SC = some concerns.

Study	D1	D2	D3	D4	D5	Overall	Basis of judgment
Navarro-Fraile et al. [14]	L	L	L	L	SC	Some concerns	Concealed block allocation and blinded assessment reported. Treating clinician unblinded, but deviations attributable to awareness were not demonstrated. The report analyzes 18/43 planned participants as a preliminary sample, rather than documenting loss of 25 participants; interim analysis/prespecification leaves D5 uncertain.
Polizzi et al. [15]	L	SC	L	L	SC	Some concerns	Computer allocation and numbered envelopes reported. Per-protocol analysis may depart from the assignment effect. All 21 enrolled patients were reported at follow-up; laboratory procedures specified. Retrospective registration limits confirmation of prespecified analyses.
Spitz et al. [17]	SC	L	L	L	SC	Some concerns	Random assignment to MOP versus no MOP reported without sequence/concealment detail. Outcomes were available for 15 paired untreated teeth and 7/8 treated teeth; RNA-seq procedures were common to groups. No prospective analysis plan identified.

B. Comparative observational studies: Newcastle–Ottawa Scale

Case–control items: S1 validated case definition; S2 representativeness; S3 community controls; S4 control definition; C comparability (0–2 stars); E1 secure/blinded exposure ascertainment; E2 same method; E3 comparable response. Cohort items (Kumar): S1 representativeness; S2 same-source unexposed group; S3 exposure ascertainment; S4 outcome absent at baseline; C comparability; O1 independent/blinded outcome assessment or record linkage; O2 adequate follow-up period; O3 follow-up completeness. ★ = awarded; – = not awarded. Item scores are newly appraised and need not reproduce the former totals.

Study	NOS form	S1	S2	S3	S4	C	E/O1	E/O2	E/O3	Total	Basis of judgment
Alhazmi et al. [21]	Case–control	★	★	–	★	0	★	★	–	5/9	Clinical/radiographic case definition; clinic rather than community controls. Genetic assay common to both groups. Broad age range; no clear adjustment for age/sex or response comparison.
Paddenberg-Schubert et al. [23]	Case–control	★	★	–	★	1	★	★	–	6/9	Consecutive clinic recruitment; defined SNB thresholds; blinded duplicate genotyping; age-adjusted regression. Clinic controls and response rates not documented separately.
Yılmaz et al. [24]	Case–control	★	–	–	★	0	★	★	–	4/9	Clinical/cephalometric diagnosis and same TaqMan assay. Clinic sample without consecutive recruitment; no matched/adjusted primary genetic association; response rates not reported.
Kumar et al. [12]	Cohort (age groups)	–	★	★	★	0	–	★	–	4/9	Convenience sample of 20 patients in two age groups; paired GCF samples before/24 h after activation. No blinded outcome assessment or adjusted between-age comparison; completion details limited.

C. Single-group before–after studies: JBI quasi-experimental checklist

Q1 temporal order; Q2 participants comparable; Q3 other care similar; Q4 separate control group; Q5 multiple measures before and after; Q6 follow-up completeness; Q7 same outcome method; Q8 measurement reliability; Q9 appropriate analysis. Y = Yes; N = No; U = Unclear. A single baseline and single later measure do not meet Q5; a baseline sample is not a separate control group under Q4.

Study	Q1	Q2	Q3	Q4	Q5	Q6	Q7	Q8	Q9	Basis of judgment
Kapoor et al. [11]	Y	Y	U	N	N	N	Y	Y	U	Five children sampled before and 9 months after appliance therapy; one follow-up serum sample missing. Growth and concurrent care cannot be separated from treatment; duplicate PCR reported.
Lepore et al. [13]	Y	Y	U	N	N	U	Y	Y	U	Six volunteers measured at baseline, 2 days and 2 weeks; only one pretreatment point and no independent group. Repeated spectroscopy documented; completion and repeated-measures analysis insufficiently clear.

Study	Q1	Q2	Q3	Q4	Q5	Q6	Q7	Q8	Q9	Basis of judgment
Velachery et al. [18]	Y	Y	U	N	N	Y	Y	Y	U	Six participants before and after debonding, with no separate comparator or plaque index. LC–MS quality control described; changes in oral hygiene/time remain plausible.
Zhang et al. [20]	Y	Y	U	N	N	Y	Y	Y	U	Thirty patients sampled at months 2 and 12, both after treatment initiation; no pretreatment or independent control. Paired biomarker measurement reported; time and treatment effects cannot be disentangled.

D. Analytical cross-sectional studies: JBI

X1 inclusion criteria; X2 subjects/setting; X3 exposure measurement; X4 objective condition definition; X5 confounders identified; X6 confounding strategies; X7 outcome measurement; X8 statistical analysis. Y = Yes; N = No; U = Unclear.

Study	X1	X2	X3	X4	X5	X6	X7	X8	Basis of judgment
Simancas-Escorcia et al. [16]	Y	Y	Y	Y	Y	Y	Y	U	Eligibility, tissue staining, gingival classification and adjusted analyses described. Cox/time-to-event claims are difficult to reconcile with a cross-sectional sample; model estimates are not prospective prediction.
Xie et al. [19]	Y	Y	Y	Y	Y	N	Y	U	Six oral-hygiene/appliance groups and standardized multi-omics methods; behavioral and oral-hygiene confounding not adequately controlled. Independence of model validation is unclear.

E. Case report: JBI

R1 demographics; R2 history/timeline; R3 presenting condition; R4 diagnostic methods/results; R5 treatment; R6 condition after treatment; R7 adverse events; R8 takeaway lessons. NA = not applicable to this diagnostic case, which does not report a study intervention.

Study	R1	R2	R3	R4	R5	R6	R7	R8	Basis of judgment
Grenga et al. [22]	Y	U	Y	Y	NA	NA	NA	Y	Ten-year-old patient and clinical/radiographic/genetic findings described; previous treatment is mentioned but longitudinal history is incomplete. No intervention or post-treatment outcome was studied; variants cannot establish causation.

Instrument sources: RoB 2 (riskofbias.info); Newcastle–Ottawa Scale (Ottawa Hospital Research Institute); JBI 2017 critical-appraisal checklists for quasi-experimental, analytical cross-sectional, and case-report designs (jbi.global).

References

Sterne, J.A.C.; Savović, J.; Page, M.J.; Elbers, R.G.; Blencowe, N.S.; Boutron, I.; Cates, C.J.; Cheng, H.-Y.; Corbett, M.S.; Eldridge, S.M.; et al. RoB 2: a revised tool for assessing risk of bias in randomised trials. BMJ 2019, 366, l4898, doi:10.1136/bmj.l4898.

Wells, G.A.; Shea, B.; O'Connell, D.; Peterson, J.; Welch, V.; Losos, M.; Tugwell, P. The Newcastle-Ottawa Scale (NOS) for Assessing the Quality of Nonrandomised Studies in Meta-Analyses. Available online: https://ohri.ca/en/who-we-are/core-facilities-and-platforms/ottawa-methods-centre/newcastle-ottawa-scale (accessed on 03/09/2026).

Tufanaru, C.; Munn, Z.; Aromataris, E.; Campbell, J.; Hopp, L. Chapter 3: Systematic Reviews of Effectiveness. In Joanna Briggs Institute Reviewer's Manual, Aromataris, E., Munn, Z., Eds.; The Joanna Briggs Institute: Adelaide, Australia, 2017.
