# Supplementary material for: Biological Variability and Biomedical Innovation in Personalized Orthodontics: A Systematic Review with Narrative Synthesis
Source: J Clin Med. 2026 Oct 9;15(20):7767. doi: 10.3390/jcm15207767 (PMC13705211; doi:10.3390/jcm15207767)
Supplement: Supplementary file 1 [file jcm-15-07767-s001.zip › Supplementary_Table_S3_Certainty_of_Evidence.pdf]

Table S3: Outcome-level certainty-of-evidence assessments and supporting judgments [14–28].

Outcome level certainty of evidence assessment for the included human studies

Certainty concerns whether the available evidence supports the stated clinical or biological conclusion. Judgments below apply GRADE principles to a narrative synthesis following Murad et al.; they are not scores assigned to individual papers. Each study addresses a distinct outcome or marker, so a single pooled estimate and between-study inconsistency assessment were generally unavailable.

Outcome and contributing study	Certainty	Reasons for judgment
Kapoor et al. [1] — salivary/serum miRNA-200a after functional-appliance therapy; n=5; no significant paired change.	Very low	Observational before–after evidence; serious risk of bias (no independent comparator, incomplete serum follow-up), very serious imprecision (five children), and indirectness to patient-level prediction. Inconsistency not assessable.
Kumar et al. [2] — GCF IL-1 after force activation and between-age difference; n=20.	Very low	Observational evidence; serious risk of bias (unadjusted age comparison and limited outcome-assessment details), serious imprecision (small age subgroups), and indirectness to treatment selection. Inconsistency not assessable.
Lepore et al. [3] — ATR-FTIR spectral change in GCF after treatment initiation; n=6.	Very low	Observational before–after evidence; serious risk of bias (no independent control), serious imprecision (six participants), and indirectness (spectral change rather than validated clinical monitoring). Inconsistency not assessable.
Navarro-Fraile et al. [4] — root resorption under individualized versus conventional force protocols; preliminary randomized sample n=18.	Very low	Randomized evidence initially rated high; downgraded for some concerns in selective reporting/interim analysis and very serious imprecision. Root-length and root-volume results differed; clinical importance and replication remain uncertain. AI measured outcomes; it did not choose forces.
Polizzi et al. [5] — GCF miRNA variation and baseline associations with three-month tooth movement; n=21.	Very low	Randomized split-mouth study initially rated high; downgraded for RoB 2 concerns (analysis/prespecification), serious imprecision (small sample and multiple markers), and indirectness to externally validated prediction. Inconsistency not assessable.
Simancas-Escorcia et al. [6]— S100A4 association with gingival enlargement and within-dataset discrimination.	Very low	Cross-sectional evidence initially rated low; downgraded for risk of bias in outcome/model interpretation and indirectness to prospective prediction. AUC=0.952 reflects the study dataset; no independent external validation was reported. Inconsistency not assessable.
Spitz et al. [7] — periodontal gene-expression response to tooth movement with or without micro-osteoperforations; n=15.	Very low	Randomized evidence initially rated high; downgraded for RoB 2 concerns (randomization/reporting), very serious imprecision (seven/eight treated participants), and indirectness of molecular expression to clinical benefit. No genes met the prespecified direct between-group threshold.
Velachery et al. [8] — salivary metabolite change following appliance removal; n=6.	Very low	Observational before–after evidence; serious risk of bias (no untreated comparator), serious imprecision (six participants and many metabolites), and indirectness to usable patient-specific monitoring. Inconsistency not assessable.
Xie et al. [9] — oral-health/appliance associations with microbiota and candidate metabolites; n=61.	Very low	Cross-sectional evidence; serious risk of bias from insufficient control of oral-hygiene and behavioral confounding and unclear independent model validation, plus indirectness to prospective clinical stratification. Inconsistency not assessable.
Zhang et al. [10] — treatment-stage salivary peptide/ApoE differences; n=30.	Very low	Observational longitudinal evidence; serious risk of bias (no pretreatment or independent control), imprecision and incomplete effect quantification, and indirectness to clinical prediction. Inconsistency not assessable.
Alhazmi et al. [11] — MSX1/PAX9/AXIN2 combinations in hypodontia and impaction; n=106.	Very low	Case–control evidence; serious risk of bias from limited confounding control, imprecision of exploratory association rules, and indirectness because treatment response was not studied. External predictive validation was not reported.

Outcome and contributing study	Certainty	Reasons for judgment
Paddenberg-Schubert et al. [12] — EGF/EGFR variation associated with mandibular retrognathism; n=119.	Very low	Case–control evidence; limitations in selection and residual confounding, imprecision of candidate genetic associations, and indirectness to treatment-response prediction. Independent replication was not established.
Yılmaz et al. [13] — MMP-1 genotype and open-bite phenotype; n=60.	Very low	Case–control evidence; serious risk of bias (unadjusted analyses and Hardy–Weinberg deviation among cases), imprecision, and indirectness to treatment response. Overall case–control genotype/allele comparisons were nonsignificant.
Grenga et al. [14] — miRNA variants and complex dental/craniofacial phenotype in one patient.	Not rated	A single descriptive case has no comparison group or association estimate. A causal genetic or predictive treatment-response claim cannot be graded from this report.

Clinical claims without direct evidence

None of the included studies tested whether AI-guided force selection improves clinical outcomes or whether genetic variants predict an individual patient's orthodontic treatment response. Certainty is not rated for these claims because direct eligible evidence is absent; this is distinct from a "very low" rating for evidence that exists.

Assessment rules and interpretation

Randomized evidence starts at high certainty and nonrandomized comparative or descriptive evidence at low certainty. Downgrading considered risk of bias, inconsistency, indirectness, imprecision, and publication bias. Inconsistency could not be assessed when only one study addressed a specific outcome. Publication bias could not be evaluated reliably for these sparse outcome groups; its absence should not be inferred. No upgrading was justified. Distinct biomarkers, phenotypes, and analytical tasks were not pooled into a single domain rating. "Very low" denotes very limited confidence that an observed effect or association will hold in a clinically relevant setting; "Not rated" denotes no comparative evidence on which to rate the stated causal claim.

Source for narrative GRADE adaptation: Murad MH, Mustafa RA, Schünemann HJ, et al. Rating the certainty in evidence in the absence of a single estimate of effect. Evidence-Based Medicine. 2017;22:85–87. doi:10.1136/ebmed-2017-110668. Study findings and numbering follow manuscript Table 2; study-level judgments follow Supplementary Table S2.

References

1. Kapoor, P.; Chowdhry, A.; Bagga, D.K.; Bhargava, D.; Katiyar, A.; Natarajan, S. Exploring the role of MicroRNA-200a in mandibular condylar cartilage growth following functional appliance therapy in growing children: A pilot study. *J. Contemp. Orthod.* **2025**, *9*, 244–251. https://doi.org/10.18231/j.jco.2025.034.
2. Kumar, B.D.; Singh, N.; Verma, S.K.; Singh, S.; Thakur, S. A Study to Evaluate Il1 And Il6 Gingival Crevicular Fluid Levels in Adolescents and Young Adults During the Early Phase of Orthodontic Tooth Movement. *J. Pharm. Bioallied Sci.* **2022**, *14*, 494–497. https://doi.org/10.4103/jpbs.jpbs_52_22.
3. Estrella, N.-F.; Alexandra, D.-S.; Yun, C.; Palma-FernÁNdez, J.C.; Alejandro, I.-L. AI-aided volumetric root resorption assessment following personalized forces in orthodontics: Preliminary results of a randomized clinical trial. *J. Evid.-Based Dent. Pract.* **2025**, *25*, 102095. https://doi.org/10.1016/j.jebdp.2025.102095.
4. Lepore, M.; Portaccio, M.; d'Apuzzo, F.; Nucci, L.; Grassia, V.; Annunziata, M.; Camerlingo, C. Speed Up Monitoring Orthodontic Treatments by Using Gingival Crevicular Fluid FT-IR Spectroscopy in ATR Geometry. *IEEE Trans. Instrum. Meas.* **2025**, *74*, 1–7. https://doi.org/10.1109/tim.2025.3565240.
5. Polizzi, A.; Alibrandi, A.; Lo Giudice, A.; Distefano, A.; Orlando, L.; Analazi, A.M.; Pizzo, G.; Volti, G.L.; Isola, G. Impact of periodontal microRNAs associated with alveolar bone remodeling during orthodontic tooth movement: A randomized clinical trial. *J. Transl. Med.* **2024**, *22*, 1–13.
6. Simancas-Escorcia, V.; Plazas-Román, J.; Díaz-Caballero, A.; Martínez-Martínez, A.; Ardila, C.M. Early prediction of orthodontic gingival enlargement using S100A4: A biomarker-based risk stratification model. *Odontology* **2026**, *114*, 1273–1282. https://doi.org/10.1007/s10266-025-01194-2.

7. Spitz, A.; Adesse, D.; Gonzalez, M.; Pellegrino, R.; Hakonarson, H.; Marañón-Vásquez, G.A.; Bolognese, A.M.; Teles, F. Effect of micro-osteoperforations on the gene expression profile of the periodontal ligament of orthodontically moved human teeth. *Clin. Oral Investig.* **2022**, *26*, 1985–1996. https://doi.org/10.1007/s00784-021-04178-y.
8. Velachery, S.; Mascarenhas, R.; Umar, D.; Ts, K.P.; Shetty, S. Tracking metabolic changes in the oral environment after multi-bracket treatment via salivary metabolomics. *Clin. Investig. Orthod.* **2025**, *84*, 253–264. https://doi.org/10.1080/27705781.2025.2567716.
9. Xie, Q.; Li, D.; Ren, C.Y.; Liang, H.; Shi, G.; Chen, W.H. Comprehensive Analysis of Orthodontic Treatment Effects on the Oral Microbiome, Metabolome, and Associated Health Indicators. *Int. Dent. J.* **2025**, *75*, 1585–1598. https://doi.org/10.1016/j.identj.2025.02.014.
10. Zhang, J.; Sun, B.; Zhao, H.; Zhang, T.; He, D.; Lin, J.; Chen, F. Apolipoprotein E is an effective biomarker for orthodontic tooth movement in patients treated with transmission straight wire appliances. *Am. J. Orthod. Dentofac. Orthop.* **2022**, *161*, 255–262.e251. https://doi.org/10.1016/j.ajodo.2020.08.020.
11. Alhazmi, N.; Alaqla, A.; Almuzzaini, B.; Aldrees, M.; Alnaqa, G.; Almasoud, F.; Thomas, S.K.; Alshamlan, H. Unlocking predictive genetic factors with artificial intelligence: Relationship between dental impaction and hypodontia evaluated via association-rule algorithms: A case–control study. *Saudi Dent. J.* **2026**, *38*, 2. https://doi.org/10.1007/s44445-025-00109-9.
12. Paddenberg-Schubert, E.; Küchler, E.; Bitencourt Reis, C.L.; Silva-Sousa, A.C.; Kirschneck, C. New insights into the genetics of mandibular retrognathism: Novel candidate genes. *J. Orofac. Orthop.* **2025**, *86*, 275–283. https://doi.org/10.1007/s00056-023-00512-z.
13. Yilmaz, Ö.; Polat, T.; Özbilen, E.; Aslan, B.T. The Influence of MMP-1 rs1799750 Gene Variant on the Severity of Open Bite Malocclusion. *Appl. Sci.* **2025**, *15*, 9786. https://doi.org/10.3390/app15179786.
14. Grenga, C.; Guarnieri, R.; Mezio, M.; De Stefano, A.A.; Galluccio, G.; Di Giorgio, R.; Giovannetti, A.; Pizzuti, A.; Caputo, V.; Barbato, E. Craniofacial and Dental Anomalies of a Patient Carrying Two MicroRNA Variants: A Proof-Of-Concept Case Report. *Clin. Case Rep.* **2025**, *13*, e70137. https://doi.org/10.1002/ccr3.70137.
