# Supplementary material for: Biological Variability and Biomedical Innovation in Personalized Orthodontics: A Systematic Review with Narrative Synthesis
Source: J Clin Med. 2026 Oct 9;15(20):7767. doi: 10.3390/jcm15207767 (PMC13705211; doi:10.3390/jcm15207767)
Supplement: Supplementary file 1 [file jcm-15-07767-s001.zip › Supplementary_Table_S4.pdf]

Table S4: Reason for article exclusion after full-text assessment [1–7,9,29–116].

Article	Reason for exclusion
Abasi, M., et al. (2025). "The Interaction Between Orthodontics and Periodontal Tissue Remodelling." Galen Medical Journal **14**.	Secondary evidence
Adel, M., et al. (2025). "Precision Assessment of Facial Asymmetry Using 3D Imaging and Artificial Intelligence." J Clin Med **14**(20).	Topic not compatible with the subject of the study
Ahmad, S. I., et al. (2026). "A Novel Multimodal Deep Image Analysis Model for Predicting Extraction/Non-Extraction Decision." Orthodontics and Craniofacial Research **29**(1): 101-111.	Topic not compatible with the subject of the study
Ahmed, N., et al. (2021). "Artificial Intelligence Techniques: Analysis, Application, and Outcome in Dentistry - A Systematic Review." Biomed Research International **2021**.	Secondary evidence
Ahn, Y., et al. (2021). "Automated mesiodens classification system using deep learning on panoramic radiographs of children." Diagnostics **11**(8).	Topic not compatible with the subject of the study
Akshaya, M., et al. (2026). "Automated dental age estimation model for panoramic radiographs." Journal of Forensic and Legal Medicine **120**.	Topic not compatible with the subject of the study
Alam, M. K., et al. (2026). "LABORATORY ASSESSMENT OF CRISPR-MEDIATED MODULATION OF OSTEOBLASTIC AND OSTEOCLASTIC GENE EXPRESSION UNDER SIMULATED ORTHODONTIC FORCE." New Armenian Medical Journal **20**(2): 11-16.	Animal, in vitro, finite element, or conceptual studies without eligible human data
Alam, M. K., et al. (2025). "Comparative Evaluation of Craniofacial Growth Patterns Using AI-Driven Longitudinal Cephalometric Superimpositions in Adolescents." J Pharm Bioallied Sci **17**(Suppl 4): S3280-s3282.	Topic not compatible with the subject of the study
Alikhani, M., et al. (2021). Precision accelerated orthodontics: How micro-osteoperforations and vibration trigger inflammation to optimize tooth movement. Biological Mechanisms of Tooth Movement**:** 265-275.	Secondary evidence
Almarhoumi, A. A. (2024). "Accuracy of Artificial Intelligence in Predicting Facial Changes Post-Orthognathic Surgery: A Comprehensive Scoping Review." Journal of Clinical and Experimental Dentistry **16**(5): e624-e633.	Secondary evidence
Almoammar, K. A. (2024). "Harnessing the Power of Artificial Intelligence in Cleft Lip and Palate: An In-Depth Analysis from Diagnosis to Treatment, a Comprehensive Review." Children-Basel **11**(2).	Secondary evidence
Alsayed, N. A. (2026). "The Role of Generative Artificial Intelligence in the Diagnosis and	Topic not compatible with the subject of the study

Treatment of Malocclusion." Cureus **18**(3): e105050.	
Anderson, M. T., et al. (2025). "Alveolar Bone Loss and Orthodontic Treatment Planning: Bone Morphology, Defects, and Force Application." Asian Journal of Periodontics and Orthodontics **5**: 185-194.	Topic not compatible with the subject of the study
Anishya, D., et al. (2025). "Neural networks-based prediction of gingival recession in camouflage-based orthodontic treatment." Journal of Contemporary Orthodontics **9**(3): 322-327.	Topic not compatible with the subject of the study
Bahrami, R. and R. Bahrami (2023). "A new model for managing tooth movement: Smart orthodontic brackets in the Internet of Dental Things (IoDT)." Medical Hypotheses **179**.	Topic not compatible with the subject of the study
Balaha, H. M., et al. (2025). "Cervical vertebral maturation assessment using an innovative artificial intelligence-based imaging analysis system." Biomedical Signal Processing and Control **100**.	Topic not compatible with the subject of the study
Balel, Y., et al. (2026). "Automated classification of sagittal, vertical, and transverse malocclusions from 3D intraoral scans via a multi-head CNN framework." Odontology.	Topic not compatible with the subject of the study
Baumann, S. P., et al. (2025). "Orthodontic Tooth Movement as a Modulator of Periodontal Bone Healing." Asian Journal of Periodontics and Orthodontics **5**: 330-340.	Topic not compatible with the subject of the study
Bhardwaj, I., et al. (2026). "Integration of Digital Twin Technology in Orthodontics: A Scoping Review." International Journal of Dentistry **2026**(1).	Secondary evidence
Cai, J. Y., et al. (2024). "Assessing the impact of occlusal plane rotation on facial aesthetics in orthodontic treatment: a machine learning approach." BMC Oral Health **24**(1).	Topic not compatible with the subject of the study
Carlson, D. S. (2015). "Evolving concepts of heredity and genetics in orthodontics." American Journal of Orthodontics and Dentofacial Orthopedics **148**(6): 922-938.	Secondary evidence
Chang, J. S., et al. (2024). "Prediction of surgery-first approach orthognathic surgery using deep learning models." International Journal of Oral and Maxillofacial Surgery **53**(11): 942-949.	Topic not compatible with the subject of the study
Chang, S., et al. (2023). "[Automated diagnostic classification with lateral cephalograms based on deep learning network model]." Zhonghua Kou Qiang Yi Xue Za Zhi **58**(6): 547-553.	Non-English article
Chen, Y., et al. (2024). "Integrated bioinformatic analysis of protein landscape in gingival crevicular fluid unveils sequential bioprocess in	Topic not compatible with the subject of the study

orthodontic tooth movement." Progress in Orthodontics **25**(1).	
Chen, Y. and C. Zhang (2023). "Role of noncoding RNAs in orthodontic tooth movement: new insights into periodontium remodeling." Journal of Translational Medicine **21**(1).	Secondary evidence
Chojnacka, K. and M. Mikulewicz (2025). "Genomic architecture of skeletal malocclusions: Implications for precision orthodontics - Narrative review." Gene Reports **41**.	Secondary evidence
Cultrera, G., et al. (2022). "MicroRNA Modulation during Orthodontic Tooth Movement: A Promising Strategy for Novel Diagnostic and Personalized Therapeutic Interventions." International Journal of Molecular Sciences **23**(24).	Secondary evidence
de Queiroz Tavares Borges Mesquita, G., et al. (2023). "Artificial Intelligence for Detecting Cephalometric Landmarks: A Systematic Review and Meta-analysis." Journal of Digital Imaging **36**(3): 1158-1179.	Secondary evidence
Dragomirescu, A. O., et al. (2025). "GENETIC ARCHITECTURES AND MOLECULAR INTERVENTIONS IN CRANIOFACIAL DEVELOPMENT: PAVING THE WAY FOR ADVANCED DIAGNOSTIC AND THERAPEUTIC FRONTIERS." Romanian Journal of Oral Rehabilitation **17**(1): 149-161.	Secondary evidence
Firth, F. A., et al. (2020). "Investigating orthodontic tooth movement: challenges and future directions." Journal of the Royal Society of New Zealand **50**(1): 67-79.	Topic not compatible with the subject of the study
Frazier-Bowers, S. A., et al. (2023). "Preface to the 9th Biennial COAST Conference: Harnessing Technology and Biomedicine for Personalized Orthodontics." Orthodontics & Craniofacial Research **26**: 4-7.	Interview/editorial/conference
Galdi, M., et al. (2026). "Sector Classification of Unerupted Maxillary Canines: A Deep Learning-Based Automated Framework Using Panoramic Radiographs." Orthodontics & Craniofacial Research.	Topic not compatible with the subject of the study
Giannini, L., et al. (2026). "Influence of Pharmacological Agents on Orthodontic Tooth Movement: A Systematic Review." Bioengineering **13**(2).	Secondary evidence
Guarnieri, R., et al. (2025). "Epigenetic Signatures of Dental Stem Cells: Insights into DNA Methylation and Noncoding RNAs." Applied Sciences (Switzerland) **15**(15).	Secondary evidence
Guo, X. C. and Y. H. Shao (2025). "AI-driven dynamic orthodontic treatment management:	Secondary evidence

personalized progress tracking and adjustments-a narrative review." Frontiers in Dental Medicine **6**.	
Gurbade, V. V., et al. (2025). Advancements in AI-Driven Cephalometric Analysis for Orthodontic Applications: A Review on Automated Landmark Detection and Angle Assessment. 4th International Conference on Sentiment Analysis and Deep Learning, ICSADL 2025 - Proceedings.	Interview/editorial/conference
Hack, M., et al. (2024). "Comparative study on the results of orthodontic diagnostics by using algorithms generated by Artificial Intelligence and simple algorithms." Medicine and Pharmacy Reports **97**(2): 215-221.	Topic not compatible with the subject of the study
Hack, M., et al. (2023). "COMPARATIVE STUDY ON THE GUIDANCE OF ORTHODONTIC TREATMENTS THROUGH ARTIFICIAL INTELLIGENCE-BASED SOFTWARE AND CLASSIC SOFTWARE." Romanian Journal of Oral Rehabilitation **15**(4): 436-448.	Topic not compatible with the subject of the study
Han, M. M., et al. (2025). "Automated Landmark Detection and Lip Thickness Classification Using a Convolutional Neural Network in Lateral Cephalometric Radiographs." Diagnostics **15**(12).	Topic not compatible with the subject of the study
Hartsfield, J. K. (2015). Personalized orthodontics: Limitations and possibilities in orthodontic practice. Biological Mechanisms of Tooth Movement: Second Edition**:** 164-172.	Secondary evidence
Hartsfield, J. K., et al. (2021). Precision orthodontics: Limitations and possibilities in practice. Biological Mechanisms of Tooth Movement**:** 189-198.	Secondary evidence
Hartsfield Jr, J. K. (2008). "Personalized Orthodontics, The Future of Genetics in Practice." Seminars in Orthodontics **14**(2): 166-171.	Secondary evidence
Hemanth, M., et al. (2015). "An Analysis of the Stress induced in the Periodontal Ligament during Extrusion and Rotation Movements- Part II: A Comparison of Linear vs Nonlinear FEM Study." J Contemp Dent Pract **16**(10): 819-823.	Animal, in vitro, finite element, or conceptual studies without eligible human data
Iber-Díaz, P., et al. (2020). "GWAS of Post-Orthodontic Aggressive External Apical Root Resorption Identified Multiple Putative Loci at X-Y Chromosomes." Journal of Personalized Medicine **10**(4).	Animal, in vitro, finite element, or conceptual studies without eligible human data
Inchingolo, F., et al. (2023). "Primary Failure Eruption: Genetic Investigation, Diagnosis and Treatment: A Systematic Review." Children-Basel **10**(11).	Secondary evidence
Iwasaki, L. R., et al. (2015). "Personalized and precision orthodontic therapy." Orthodontics & Craniofacial Research **18**: 1-7.	Secondary evidence

Janjic, K., et al. (2023). "Differential gene expression and protein-protein interaction networks of human periodontal ligament stromal cells under mechanical tension." European Journal of Cell Biology **102**(2).	Animal, in vitro, finite element, or conceptual studies without eligible human data
Jheon, A. H., et al. (2017). "Moving towards precision orthodontics: An evolving paradigm shift in the planning and delivery of customized orthodontic therapy." Orthodontics & Craniofacial Research **20**: 106-113.	Topic not compatible with the subject of the study
Johansson, L. M., et al. (2024). "Biomechanical Adaptation of the Periodontium to Orthodontic Forces: Insights from Computational and Theoretical Studies." Asian Journal of Periodontics and Orthodontics **4**: 265-274.	Secondary evidence
Junaid, N., et al. (2022). "Development, Application, and Performance of Artificial Intelligence in Cephalometric Landmark Identification and Diagnosis: A Systematic Review." Healthcare **10**(12).	Secondary evidence
Kapila, S., et al. (2023). "Connecting the dots towards precision orthodontics." Orthodontics & Craniofacial Research **26**: 8-19.	Secondary evidence
Kapoor, P., et al. (2021). "MicroRNAs in oral fluids (saliva and gingival crevicular fluid) as biomarkers in orthodontics: systematic review and integrated bioinformatic analysis." Progress in Orthodontics **22**(1).	Secondary evidence
Kapoor, P., et al. (2014). "Effect of orthodontic forces on cytokine and receptor levels in gingival crevicular fluid: a systematic review." Prog Orthod **15**(1): 65.	Secondary evidence
Khanagar, S. B., et al. (2021). "Scope and performance of artificial intelligence technology in orthodontic diagnosis, treatment planning, and clinical decision-making - A systematic review." Journal of Dental Sciences **16**(1): 482-492.	Secondary evidence
Krishnan, V. and Z. Davidovitch (2021). Biological basis of orthodontic tooth movement: A historical perspective. Biological Mechanisms of Tooth Movement**:** 3-15.	Secondary evidence
Krishnan, V., et al. (2021). Planning and executing tooth-movement research. Biological Mechanisms of Tooth Movement**:** 311-326.	Secondary evidence
Krishnan, V., et al. (2021). Controversies and research directions in toothmovement research. Biological Mechanisms of Tooth Movement**:** 327-342.	Secondary evidence
Kumar, S., et al. (2026). The use of AI-based predictive models in personalized treatment	Interview/editorial/conference

plans for orthodontics. AIP Conference Proceedings.	
Kuroda, T. (2020). "Evidence-based individualized orthodontic treatment: The future of orthodontics?" Journal of the World Federation of Orthodontists **9**(4): 139-145.	Secondary evidence
La Rosa, S., et al. (2026). "Orthodontists' Perceptions, Attitudes, and Adoption of Artificial Intelligence: A Scoping Review." Dent J (Basel) **14**(5).	Secondary evidence
Lin, X., et al. (2026). "The frontiers of orthodontically induced inflammatory root resorption: mechanism, treatment, and future prospects." J Endod.	Secondary evidence
Lone, I. M., et al. (2023). "Anterior Open Bite Malocclusion: From Clinical Treatment Strategies towards the Dissection of the Genetic Bases of the Disease Using Human and Collaborative Cross Mice Cohorts." Journal of Personalized Medicine **13**(11)	Topic not compatible with the subject of the study
Maltha, J. C. and A. M. Kuijpers-Jagtman (2023). "Mechanobiology of orthodontic tooth movement: An update." Journal of the World Federation of Orthodontists **12**(4): 156-160.	Secondary evidence
McLean, S. L., et al. (2025). "Integrating Molecular Biology and Biomechanics in Orthodontic Treatment of Periodontitis." Asian Journal of Periodontics and Orthodontics **5**: 214-224.	Secondary evidence
Miranda, F., et al. (2023). "Artificial Intelligence Applications in Orthodontics." Journal of the California Dental Association **51**(1).	Secondary evidence
Modafferi, C., et al. (2025). "Syndromic and Non-Syndromic Primary Failure of Tooth Eruption: A Genetic Overview." Genes **16**(2).	Secondary evidence
Mostafa, Y. A., et al. (1983). "Orchestration of tooth movement." American Journal of Orthodontics **83**(3): 245-250.	Secondary evidence
Niederau, C., et al. (2025). "Jaw-specific differential kinase activity profiles in human periodontal ligament stem cells under mechanical compression." Stem Cell Research and Therapy **16**(1).	Topic not compatible with the subject of the study
Nitzsche, A., et al. (2024). "GDF15 Modulates the Zoledronic-Acid-Induced Hyperinflammatory Mechanoresponse of Periodontal Ligament Fibroblasts." Cells **13**(2).	Topic not compatible with the subject of the study
Özden, S., et al. (2026). "Automated deep learning detection of orthodontically induced external apical root resorption in maxillary incisors on panoramic radiographs." Progress in Orthodontics **27**(1).	Topic not compatible with the subject of the study

Pacheco-Quito, E. M., et al. (2026). "Pharmacological Modulation of Orthodontic Tooth Movement: Mechanisms, Clinical Implications, and Emerging Therapeutic Approaches. A Review." Clinical Cosmetic and Investigational Dentistry **18**.	Secondary evidence
Papagiannis, A., et al. (2021). "Orthodontic Treatment of a Patient with Dentin Dysplasia Type I and Bilateral Maxillary Canine Impaction: Case Presentation and a Family-Based Genetic Analysis." Children-Basel **8**(6).	Topic not compatible with the subject of the study
Pinheiro, L. H. M., et al. (2021). "Genetic variation involved in the risk to external apical root resorption in orthodontic patients: a systematic review." Clinical Oral Investigations **25**(10): 5613-5627.	Secondary evidence
Pniak, M. (2024). "Hemifacial microsomia – a review of the literature of current hypotheses of pathogenesis, and possible therapeutic methods." Polish Otorhinolaryngology Review **13**(3): 14-21.	Topic not compatible with the subject of the study
Proffit, W. R. and S. A. Frazier-Bowers (2009). "Mechanism and control of tooth eruption: Overview and clinical implications." Orthodontics and Craniofacial Research **12**(2): 59-66.	Secondary evidence
Qin, X., et al. (2019). "The circadian expression of osteogenic factors in periodontal tissue loading mechanical force: new concepts of the personalized orthodontic care." Epma Journal **10**(1).	Animal, in vitro, finite element, or conceptual studies without eligible human data
S., L. R., et al. (2025). "The impact of artificial intelligence on orthodontics: a systematic review of applications and implications." Minerva Dent Oral Sci **74**(3): 195-208.	Secondary evidence
Sadeq, S. M. A., et al. (2025). "The Use of Salivary and Gingival Crevicular Fluid Biomarkers in Predicting Orthodontic Treatment Response." Clinical Cosmetic and Investigational Dentistry **17**: 499-513.	Secondary evidence
Sagar, S., et al. (2025). "Decoding the Biochemical Pathways of Orthodontic Tooth Movement: A Focus on Salivary IL-17A and 1,25-Dihydroxycholecalciferol." Texila International Journal of Public Health **13**(2).	Secondary evidence
Schmidt, F. and B. G. Lapatki (2019). "Effect of variable periodontal ligament thickness and its non-linear material properties on the location of a tooth's centre of resistance." Journal of Biomechanics **94**: 211-218.	Animal, in vitro, finite element, or conceptual studies without eligible human data
Schuldt, L., et al. (2022). "Oleic acid-related anti-inflammatory effects in force-stressed PdL fibroblasts are mediated by H3 lysine acetylation	Animal, in vitro, finite element, or conceptual studies without eligible human data

associated with altered IL10 expression." Epigenetics **17**(13): 1892-1904.	
Sharma, S., et al. (2026). "Unveiling molecular dynamics: the effects of functional mandibular advancement and a one-month recovery period on myostatin and myosin-1c gene expression in masticatory muscles of young male Wistar rats." Folia Medica **68**(2).	Animal, in vitro, finite element, or conceptual studies without eligible human data
Smith, R. J. and C. J. Burstone (1984). "Mechanics of tooth movement." American Journal of Orthodontics **85**(4): 294-307.	Secondary evidence
Sobhani, M., et al. (2025). "Biological Interplay between Orthodontic Forces and Apical Papilla-derived Stem Cells: A Review on Mechanisms and Implications." Galen Med J **14**(S Pt 1): e3946.	Secondary evidence
Steinberg, T., et al. (2025). "Shaping Orthodontics of the Future: Concepts and Implications from a Cellular and Molecular Perspective." International Journal of Molecular Sciences **26**(17).	Secondary evidence
Tang, Z. and Y. Yang (2026). "Mechanobiology in orthodontic tooth movement: update on knowledge and clinical implications." Seminars in Orthodontics **32**(2): 338-347.	Secondary evidence
Utreja, A. (2018). "Low-Magnitude Forces for Bone Modeling and Remodeling in Dentofacial Orthopedics." Current Osteoporosis Reports **16**(3): 277-282.	Secondary evidence
van Dijk, P. J., et al. (2025). "Interdisciplinary Orthodontic–Periodontal Management in Adult Patients: Timing, Sequencing, and Risk Control." Asian Journal of Periodontics and Orthodontics **5**: 246-256.	Topic not compatible with the subject of the study
Vora, S. R., et al. (2025). "Preface to the 10th Biennial COAST Conference: AI- and Biomedicine-Driven Precision Orthodontics and Craniofacial Care." Orthodontics & Craniofacial Research **28**: S4-S9.	Interview/editorial/conference
Wang, Y. F., et al. (2026). "Biological Determinants and non-pharmacological management of orthodontic pain." Iscience **29**(6).	Topic not compatible with the subject of the study
Weber, A. K., et al. (2025). "Integrating Periodontal Phenotype in Orthodontic Force and Timing: A Framework." Asian Journal of Periodontics and Orthodontics **5**: 277-287.	Topic not compatible with the subject of the study
Yadalam, P. K., et al. (2026). AI-Driven Stratification of Gingival Inflammation Progression During Orthodontic Aligner Therapy: A Dynamic Modeling Approach. Proceedings of the 2026 12th International Conference on	Interview/editorial/conference

Communication and Signal Processing, ICCSP 2026.	
Yassir, Y. A., et al. (2021). "Orthodontic treatment and root resorption: an overview of systematic reviews." European Journal of Orthodontics **43**(4): 442-456.	Secondary evidence
Zhang, J., et al. (2020). "Analysis of salivany peptidomics during orthodontic treatment." Chinese Journal of Orthodontics **27**(1): 45-48.	Secondary evidence
Zhang, M., et al. (2023). "Digital working process in diagnosis, treatment planning and fabrication of personalized orthodontic appliances." Digital Medicine **9**(2).	Non-English article
Zhou, Y. K., et al. (2026). "Mechanical forces orchestrate the epigenetic landscape of oral mesenchymal stem/progenitor cell fate in dental and periodontal tissues." Frontiers in Cell and Developmental Biology **14**.	Topic not compatible with the subject of the study

References

1. Abasi, M.; Goodarzi, A.; Solhmirzaei, R.; Fazel, F.; Moharrami, A.; Ghasemi, H.; Milani, H.A. The Interaction Between Orthodontics and Periodontal Tissue Remodeling. Galen Medical Journal 2025, 14, doi:10.31661/gmj.v14i.4005.
2. Adel, M.; Hunt, K.J.; Lau, D.; Hartsfield, J.K.; Reyes-Centeno, H.; Beeman, C.S.; Elshebiny, T.; Sharab, L. Precision Assessment of Facial Asymmetry Using 3D Imaging and Artificial Intelligence. J Clin Med 2025, 14, doi:10.3390/jcm14207172.
3. Ahmad, S.I.; Olczyk, J.; Araújo, A.S.; de Moura Medeiros, J.P.; Teixeira, V.C.; Gomes, C.F.A.; Magnaguagno, M.C.; Roederer, Q.; Dutra, V.; Conley, R.S.; et al. A Novel Multimodal Deep Image Analysis Model for Predicting Extraction/Non-Extraction Decision. Orthodontics and Craniofacial Research 2026, 29, 101-111, doi:10.1111/ocr.70057.
4. Ahmed, N.; Abbasi, M.S.; Zuberi, F.; Qamar, W.; Halim, M.S.B.; Maqsood, A.; Alam, M.K. Artificial Intelligence Techniques: Analysis, Application, and Outcome in Dentistry - A Systematic Review. BioMed Research International 2021, 2021, doi:10.1155/2021/9751564.
5. Ahn, Y.; Hwang, J.; Jung, Y.H.; Jeong, T.; Shin, J. Automated mesiodens classification system using deep learning on panoramic radiographs of children. Diagnostics 2021, 11, doi:10.3390/diagnostics11081477.
6. Akshaya, M.; Vijayalakshmi, K.R.; Kumar, T.N.R.; Rakshana, M. Automated dental age estimation model for panoramic radiographs. Journal of Forensic and Legal Medicine 2026, 120, doi:10.1016/j.jflm.2026.103148.
7. Alam, M.K.; Almohammed, Y.E.M.; Hajeer, M.Y.; Alanazi, A.W.N.; Alanazi, F.S.A.; Alnawmasi, Y.M.F. LABORATORY ASSESSMENT OF CRISPR-MEDIATED MODULATION OF OSTEOBLASTIC AND OSTEOCLASTIC GENE EXPRESSION UNDER SIMULATED ORTHODONTIC FORCE. New Armenian Medical Journal 2026, 20, 11-16, doi:10.56936/18290825-2026.20v.2-11.
8. Alam, M.K.; Hajeer, M.Y.; Abahussain, R.N.; Algadri, R.A.; Alamri, D.M.; Rashid, M.E. Comparative Evaluation of Craniofacial Growth Patterns Using AI-Driven Longitudinal Cephalometric Superimpositions in Adolescents. J Pharm Bioallied Sci 2025, 17, S3280-s3282, doi:10.4103/jpbs.jpbs_1346_25.
9. Alikhani, M.; Nervina, J.M.; Teixeira, C.C. Precision accelerated orthodontics: How micro-osteoperforations and vibration trigger inflammation to optimize tooth movement. In Biological Mechanisms of Tooth Movement; 2021; pp. 265-275.
10. Almarhoumi, A.A. Accuracy of Artificial Intelligence in Predicting Facial Changes Post-Orthognathic Surgery: A Comprehensive Scoping Review. Journal of Clinical and Experimental Dentistry 2024, 16, e624-e633, doi:10.4317/jced.61500.

11. Almoammar, K.A. Harnessing the Power of Artificial Intelligence in Cleft Lip and Palate: An In-Depth Analysis from Diagnosis to Treatment, a Comprehensive Review. Children-Basel 2024, 11, doi:10.3390/children11020140.
12. Alsayed, N.A. The Role of Generative Artificial Intelligence in the Diagnosis and Treatment of Malocclusion. Cureus 2026, 18, e105050, doi:10.7759/cureus.105050.
13. Anderson, M.T.; Petrenko, O.V.; Ho, P.J. Alveolar Bone Loss and Orthodontic Treatment Planning: Bone Morphology, Defects, and Force Application. Asian Journal of Periodontics and Orthodontics 2025, 5, 185-194, doi:10.51847/zg4TjG0uxO.
14. Anishya, D.; Kumar, P.; Arvind, P. Neural networks-based prediction of gingival recession in camouflage-based orthodontic treatment. Journal of Contemporary Orthodontics 2025, 9, 322-327, doi:10.18231/j.jco.v.9.i.3.5.
15. Bahrami, R.; Bahrami, R. A new model for managing tooth movement: Smart orthodontic brackets in the Internet of Dental Things (IoDT). Medical Hypotheses 2023, 179, doi:10.1016/j.mehy.2023.111168.
16. Balaha, H.M.; Alksas, A.; Fattal, A.; Sewelam, A.A.; Aboelmaaty, W.; Abdel-Ghaffar, K.; Deguchi, T.; El-Baz, A. Cervical vertebral maturation assessment using an innovative artificial intelligence-based imaging analysis system. Biomedical Signal Processing and Control 2025, 100, doi:10.1016/j.bspc.2024.107088.
17. Balel, Y.; Sağtaş, K.; Teke, F.; Kurt, M.A. Automated classification of sagittal, vertical, and transverse malocclusions from 3D intraoral scans via a multi-head CNN framework. Odontology 2026, doi:10.1007/s10266-026-01400-9.
18. Baumann, S.P.; Bekele, T.M.; Romano, L.F. Orthodontic Tooth Movement as a Modulator of Periodontal Bone Healing. Asian Journal of Periodontics and Orthodontics 2025, 5, 330-340, doi:10.51847/zdM4edrNrt.
19. Bhardwaj, I.; Jose, N.P.; Nambiar, S.; Ahuja, D.; Rao, S.; Prasad, S. Integration of Digital Twin Technology in Orthodontics: A Scoping Review. International Journal of Dentistry 2026, 2026, doi:10.1155/ijod/3347380.
20. Cai, J.Y.; Min, Z.Y.; Deng, Y.D.; Jing, D.; Zhao, Z.H. Assessing the impact of occlusal plane rotation on facial aesthetics in orthodontic treatment: a machine learning approach. Bmc Oral Health 2024, 24, doi:10.1186/s12903-023-03817-y.
21. Carlson, D.S. Evolving concepts of heredity and genetics in orthodontics. *American Journal of Orthodontics and Dentofacial Orthopedics* **2015**, *148*, 922-938, doi:10.1016/j.ajodo.2015.09.012.
22. Chang, J.S.; Ma, C.Y.; Ko, E.W.C. Prediction of surgery-first approach orthognathic surgery using deep learning models. International Journal of Oral and Maxillofacial Surgery 2024, 53, 942-949, doi:10.1016/j.ijom.2024.05.003.
23. Chang, S.; Wang, S.F.; Zuo, F.F.; Wang, F.; Gong, B.W.; Wang, Y.J.; Xie, X.J. [Automated diagnostic classification with lateral cephalograms based on deep learning network model]. Zhonghua Kou Qiang Yi Xue Za Zhi 2023, 58, 547-553, doi:10.3760/cma.j.cn112144-20230305-00072.
24. Chen, Y.; Mei, L.; Qian, Y.R.; Zhou, X.L.Y.; Zhao, Z.H.; Zheng, W.; Li, Y. Integrated bioinformatic analysis of protein landscape in gingival crevicular fluid unveils sequential bioprocess in orthodontic tooth movement. *Progress in Orthodontics* **2024**, *25*, doi:10.1186/s40510-024-00536-0.
25. Chen, Y.; Zhang, C. Role of noncoding RNAs in orthodontic tooth movement: new insights into periodontium remodeling. *Journal of Translational Medicine* **2023**, *21*, doi:10.1186/s12967-023-03951-9.
26. Chojnacka, K.; Mikulewicz, M. Genomic architecture of skeletal malocclusions: Implications for precision orthodontics - Narrative review. Gene Reports 2025, 41, doi:10.1016/j.genrep.2025.102347.
27. Cultrera, G.; Lo Giudice, A.; Santonocito, S.; Ronsivalle, V.; Conforte, C.; Reitano, G.; Leonardi, R.; Isola, G. MicroRNA Modulation during Orthodontic Tooth Movement: A Promising Strategy for Novel Diagnostic and Personalized Therapeutic Interventions. International Journal of Molecular Sciences 2022, 23, doi:10.3390/ijms232415501.
28. de Queiroz Tavares Borges Mesquita, G.; Vieira, W.A.; Vidigal, M.T.C.; Travençolo, B.A.N.; Beaini, T.L.; Spin-Neto, R.; Paranhos, L.R.; de Brito Júnior, R.B. Artificial Intelligence for Detecting Cephalometric Landmarks: A Systematic Review and Meta-analysis. Journal of Digital Imaging 2023, 36, 1158-1179, doi:10.1007/s10278-022-00766-w.
29. Dragomirescu, A.O.; Balua, A.M.; Albu, S.D.; Tanase, M.; Merisescu, M.M.; Albu, C.C.; Ionescu, E. GENETIC ARCHITECTURES AND MOLECULAR INTERVENTIONS IN CRANIOFACIAL DEVELOPMENT: PAVING THE WAY FOR ADVANCED DIAGNOSTIC AND THERAPEUTIC FRONTIERS. Romanian Journal of Oral Rehabilitation 2025, 17, 149-161, doi:10.62610/rjor.2025.1.17.16.
30. Firth, F.A.; Farrar, R.; Farella, M. Investigating orthodontic tooth movement: challenges and future directions. Journal of the Royal Society of New Zealand 2020, 50, 67-79, doi:10.1080/03036758.2019.1684957.
31. Frazier-Bowers, S.A.; Allareddy, V.; Venugopalan, S.R.; Lamani, E.; Vora, S.R.; Kapila, S. Preface to the 9th Biennial COAST Conference: Harnessing Technology and Biomedicine for Personalized Orthodontics. Orthodontics & Craniofacial Research 2023, 26, 4-7, doi:10.1111/ocr.12681.

32. Galdi, M.; Cannatà, D.; Celentano, F.; Rizzo, L.; Rossi, D.; Bocchino, T.; Tortora, G.; Martina, S. Sector Classification of Unerupted Maxillary Canines: A Deep Learning-Based Automated Framework Using Panoramic Radiographs. Orthodontics & Craniofacial Research 2026, doi:10.1111/ocr.70106.
33. Giannini, L.; Macrì, F.; Inchingolo, A.M.; Inchingolo, F.; Dipalma, G.; Maspero, C. Influence of Pharmacological Agents on Orthodontic Tooth Movement: A Systematic Review. Bioengineering 2026, 13, doi:10.3390/bioengineering13020224.
34. Guarnieri, R.; Giovannetti, A.; Marigliani, G.; Pieroni, M.; Mazza, T.; Barbato, E.; Caputo, V. Epigenetic Signatures of Dental Stem Cells: Insights into DNA Methylation and Noncoding RNAs. Applied Sciences (Switzerland) 2025, 15, doi:10.3390/app15158749.
35. Guo, X.C.; Shao, Y.H. AI-driven dynamic orthodontic treatment management: personalized progress tracking and adjustments-a narrative review. Frontiers in Dental Medicine 2025, 6, doi:10.3389/fdmed.2025.1612441.
36. Gurbade, V.V.; Pacharaaney, U.; Puri, C. Advancements in AI-Driven Cephalometric Analysis for Orthodontic Applications: A Review on Automated Landmark Detection and Angle Assessment. In Proceedings of the 4th International Conference on Sentiment Analysis and Deep Learning, ICSADL 2025 - Proceedings, 2025; pp. 1731-1736.
37. Hack, M.; Drăgulin, B.; Hack, L.; ElSaafin, M.; Dumitrescu, I.; Stan, D.; Păcurar, M. Comparative study on the results of orthodontic diagnostics by using algorithms generated by Artificial Intelligence and simple algorithms. Medicine and Pharmacy Reports 2024, 97, 215-221, doi:10.15386/mpr-2702.
38. Hack, M.; Hack, L.; Delean, A.; Bucur, S.; Nedelcu, E.; Dragulin, B.; Stan, D.; Gruia, V.; Pacurar, M. COMPARATIVE STUDY ON THE GUIDANCE OF ORTHODONTIC TREATMENTS THROUGH ARTIFICIAL INTELLIGENCE-BASED SOFTWARE AND CLASSIC SOFTWARE. Romanian Journal of Oral Rehabilitation 2023, 15, 436-448.
39. Han, M.M.; Huo, Z.Q.; Ren, J.Y.; Zhu, H.T.; Li, H.; Li, J.L.; Mei, L. Automated Landmark Detection and Lip Thickness Classification Using a Convolutional Neural Network in Lateral Cephalometric Radiographs. Diagnostics 2025, 15, doi:10.3390/diagnostics15121468.
40. Hartsfield, J.K. Personalized orthodontics: Limitations and possibilities in orthodontic practice. In Biological Mechanisms of Tooth Movement: Second Edition; 2015; pp. 164-172.
41. Hartsfield, J.K.; Gudsoorkar, P.; Morford, L.A. Precision orthodontics: Limitations and possibilities in practice. In Biological Mechanisms of Tooth Movement; 2021; pp. 189-198.
42. Hartsfield Jr, J.K. Personalized Orthodontics, The Future of Genetics in Practice. Seminars in Orthodontics 2008, 14, 166-171, doi:10.1053/j.sodo.2008.02.007.
43. Hemanth, M.; Raghuveer, H.P.; Rani, M.S.; Hegde, C.; Kabbur, K.J.; Chaithra, D.; Vedavathi, B. An Analysis of the Stress induced in the Periodontal Ligament during Extrusion and Rotation Movements- Part II: A Comparison of Linear vs Nonlinear FEM Study. J Contemp Dent Pract 2015, 16, 819-823, doi:10.5005/jp-journals-10024-1763.
44. Iber-Díaz, P.; Senen-Carramolino, R.; Iglesias-Linares, A.; Fernández-Navarro, P.; Flores-Mir, C.; Yañez-Vico, R.M. GWAS of Post-Orthodontic Aggressive External Apical Root Resorption Identified Multiple Putative Loci at X-Y Chromosomes. Journal of Personalized Medicine 2020, 10, doi:10.3390/jpm10040169.
45. Inchingolo, F.; Ferrara, I.; Viapiano, F.; Ciocia, A.M.; Palumbo, I.; Guglielmo, M.; Inchingolo, A.D.; Palermo, A.; Bordea, I.R.; Inchingolo, A.M.; et al. Primary Failure Eruption: Genetic Investigation, Diagnosis and Treatment: A Systematic Review. Children-Basel 2023, 10, doi:10.3390/children10111781.
46. Iwasaki, L.R.; Covell, D.A.; Frazier-Bowers, S.A.; Kapila, S.; Huja, S.S.; Nickel, J.C. Personalized and precision orthodontic therapy. Orthodontics & Craniofacial Research 2015, 18, 1-7, doi:10.1111/ocr.12089.
47. Janjic, K.; Nemec, M.; Maaser, J.L.; Sagl, B.; Jonke, E.; Andrukhov, O. Differential gene expression and protein-protein interaction networks of human periodontal ligament stromal cells under mechanical tension. European Journal of Cell Biology 2023, 102, doi:10.1016/j.ejcb.2023.151319.
48. Jheon, A.H.; Oberoi, S.; Solem, R.C.; Kapila, S. Moving towards precision orthodontics: An evolving paradigm shift in the planning and delivery of customized orthodontic therapy. Orthodontics & Craniofacial Research 2017, 20, 106-113, doi:10.1111/ocr.12171.
49. Johansson, L.M.; Svensson, E.; Olofsson, K.; Lindgren, S. Biomechanical Adaptation of the Periodontium to Orthodontic Forces: Insights from Computational and Theoretical Studies. Asian Journal of Periodontics and Orthodontics 2024, 4, 265-274, doi:10.51847/kjXnPm2sAh.

50. Junaid, N.; Khan, N.; Ahmed, N.; Abbasi, M.S.; Das, G.; Maqsood, A.; Ahmed, A.R.; Marya, A.; Alam, M.K.; Heboyan, A. Development, Application, and Performance of Artificial Intelligence in Cephalometric Landmark Identification and Diagnosis: A Systematic Review. Healthcare 2022, 10, doi:10.3390/healthcare10122454.
51. Kapila, S.; Vora, S.R.; Venugopalan, S.R.; Elnagar, M.H.; Akyalcin, S. Connecting the dots towards precision orthodontics. Orthodontics & Craniofacial Research 2023, 26, 8-19, doi:10.1111/ocr.12725.
52. Kapoor, P.; Kharbanda, O.P.; Monga, N.; Miglani, R.; Kapila, S. Effect of orthodontic forces on cytokine and receptor levels in gingival crevicular fluid: a systematic review. *Prog Orthod* **2014**, *15*, 65, doi:10.1186/s40510-014-0065-6.
53. Kapoor, P.; Chowdhry, A.; Bagga, D.K.; Bhargava, D.; Aishwarya, S. MicroRNAs in oral fluids (saliva and gingival crevicular fluid) as biomarkers in orthodontics: systematic review and integrated bioinformatic analysis. Progress in Orthodontics 2021, 22, doi:10.1186/s40510-021-00377-1.
54. Khanagar, S.B.; Al-Ehaideb, A.; Vishwanathaiah, S.; Maganur, P.C.; Patil, S.; Naik, S.; Baeshen, H.A.; Sarode, S.S. Scope and performance of artificial intelligence technology in orthodontic diagnosis, treatment planning, and clinical decision-making - A systematic review. Journal of Dental Sciences 2021, 16, 482-492, doi:10.1016/j.jds.2020.05.022.
55. Krishnan, V.; Davidovitch, Z. Biological basis of orthodontic tooth movement: A historical perspective. In Biological Mechanisms of Tooth Movement; 2021; pp. 3-15.
56. Krishnan, V.; Davidovitch, Z.; Ramachandran, R. Planning and executing tooth-movement research. In Biological Mechanisms of Tooth Movement; 2021; pp. 311-326.
57. Krishnan, V.; Kuijpers-Jagtman, A.M.; Davidovitch, Z. Controversies and research directions in toothmovement research. In Biological Mechanisms of Tooth Movement; 2021; pp. 327-342.
58. Kumar, S.; Jadhav, S.; Mali, C.N.; Karwa, P.N.; Kant, S. The use of AI-based predictive models in personalized treatment plans for orthodontics. In Proceedings of the AIP Conference Proceedings, 2026.
59. Kuroda, T. Evidence-based individualized orthodontic treatment: The future of orthodontics? Journal of the World Federation of Orthodontists 2020, 9, 139-145, doi:10.1016/j.ejwf.2020.11.001.
60. La Rosa, S.; Nucci, L.; Grippaudo, C.; Leonardi, R.; Polizzi, A. Orthodontists' Perceptions, Attitudes, and Adoption of Artificial Intelligence: A Scoping Review. Dent J (Basel) 2026, 14, doi:10.3390/dj14050257.
61. Lin, X.; Chen, S.; Cai, J.; Cai, J.; Chen, L.; Chen, Z.; Huang, P.; Chen, Z.; Cai, S.; Xu, L.; et al. The frontiers of orthodontically induced inflammatory root resorption: mechanism, treatment, and future prospects. J Endod 2026, doi:10.1016/j.joen.2026.06.005.
62. Lone, I.M.; Zohud, O.; Midlej, K.; Paddenberg, E.; Krohn, S.; Kirschneck, C.; Proff, P.; Watted, N.; Iraqi, F.A. Anterior Open Bite Malocclusion: From Clinical Treatment Strategies towards the Dissection of the Genetic Bases of the Disease Using Human and Collaborative Cross Mice Cohorts. Journal of Personalized Medicine 2023, 13, doi:10.3390/jpm13111617.
63. Maltha, J.C.; Kuijpers-Jagtman, A.M. Mechanobiology of orthodontic tooth movement: An update. *Journal of the World Federation of Orthodontists* **2023**, *12*, 156-160, doi:https://doi.org/10.1016/j.ejwf.2023.05.001.
64. McLean, S.L.; Seok, K.M.; Saleh, D.M. Integrating Molecular Biology and Biomechanics in Orthodontic Treatment of Periodontitis. Asian Journal of Periodontics and Orthodontics 2025, 5, 214-224, doi:10.51847/beaJra5evX.
65. Miranda, F.; Barone, S.; Gillot, M.; Baquero, B.; Anchling, L.; Hutin, N.; Gurgel, M.; Al Turkestani, N.; Huang, Y.; Massaro, C.; et al. Artificial Intelligence Applications in Orthodontics. Journal of the California Dental Association 2023, 51, doi:10.1080/19424396.2023.2195585.
66. Modafferi, C.; Tabolacci, E.; Grippaudo, C.; Chiurazzi, P. Syndromic and Non-Syndromic Primary Failure of Tooth Eruption: A Genetic Overview. Genes 2025, 16, doi:10.3390/genes16020147.
67. Mostafa, Y.A.; Weaks-Dybvig, M.; Osdoby, P. Orchestration of tooth movement. American Journal of Orthodontics 1983, 83, 245-250, doi:10.1016/0002-9416(83)90088-X.
68. Niederau, C.; Maas, S.L.; van der Vorst, E.P.C.; Schurgers, L.J.; Shi, Y.; Hölzle, F.; Wolf, M.; Craveiro, R.B. Jaw-specific differential kinase activity profiles in human periodontal ligament stem cells under mechanical compression. Stem Cell Research and Therapy 2025, 16, doi:10.1186/s13287-025-04778-5.
69. Nitzsche, A.; Hennig, C.L.; von Brandenstein, K.; Döding, A.; Schulze-Späte, U.; Symmank, J.; Jacobs, C. GDF15 Modulates the Zoledronic-Acid-Induced Hyperinflammatory Mechanoresponse of Periodontal Ligament Fibroblasts. Cells 2024, 13, doi:10.3390/cells13020147.

70. Özden, S.; Kula, B.; Tankus, M. Automated deep learning detection of orthodontically induced external apical root resorption in maxillary incisors on panoramic radiographs. Progress in Orthodontics 2026, 27, doi:10.1186/s40510-026-00610-9.
71. Pacheco-Quito, E.M.; Flores-Regalado, C.; Lima-Illescas, M.; Sarmiento-Ordoñez, J.; Cuenca-León, K. Pharmacological Modulation of Orthodontic Tooth Movement: Mechanisms, Clinical Implications, and Emerging Therapeutic Approaches. A Review. Clinical Cosmetic and Investigational Dentistry 2026, 18, doi:10.2147/ccide.S595185.
72. Papagiannis, A.; Fanourakis, G.; Mitsea, A.; Karayianni, K.; Vastardis, H.; Sifakakis, I. Orthodontic Treatment of a Patient with Dentin Dysplasia Type I and Bilateral Maxillary Canine Impaction: Case Presentation and a Family-Based Genetic Analysis. Children-Basel 2021, 8, doi:10.3390/children8060519.
73. Pinheiro, L.H.M.; Guimarães, L.S.; Antunes, L.S.; Küchler, E.C.; Kirschneck, C.; Antunes, L.A.A. Genetic variation involved in the risk to external apical root resorption in orthodontic patients: a systematic review. Clinical Oral Investigations 2021, 25, 5613-5627, doi:10.1007/s00784-021-04074-5.
74. Pniak, M. Hemifacial microsomia – a review of the literature of current hypotheses of pathogenesis, and possible therapeutic methods. Polish Otorhinolaryngology Review 2024, 13, 14-21, doi:10.5604/01.3001.0054.7072.
75. Proffit, W.R.; Frazier-Bowers, S.A. Mechanism and control of tooth eruption: Overview and clinical implications. Orthodontics and Craniofacial Research 2009, 12, 59-66, doi:10.1111/j.1601-6343.2009.01438.x.
76. Qin, X.; Li, Q.L.; Chen, W.M.; Bai, Y.M.; Baban, B.; Mao, J. The circadian expression of osteogenic factors in periodontal tissue loading mechanical force: new concepts of the personalized orthodontic care. Epma Journal 2019, 10, doi:10.1007/s13167-019-0161-2.
77. S., L.R.; Uzunçibuk, H.; Almeida, L.E.; Meto, A.; Veeraraghavan, V.P.; Heboyan, A. The impact of artificial intelligence on orthodontics: a systematic review of applications and implications. *Minerva Dent Oral Sci* **2025**, *74*, 195-208, doi:10.23736/s2724-6329.24.04930-1.
78. Sadeq, S.M.A.; Al Ansari, N.; Kadhem, Z.K.; Hussein, H.M. The Use of Salivary and Gingival Crevicular Fluid Biomarkers in Predicting Orthodontic Treatment Response. Clinical Cosmetic and Investigational Dentistry 2025, 17, 499-513, doi:10.2147/ccide.S552825.
79. Sagar, S.; Ramani, P.; Jeyaraj, G.; Jayaraman, S.; Moses, S. Decoding the Biochemical Pathways of Orthodontic Tooth Movement: A Focus on Salivary IL-17A and 1,25-Dihydroxycholecalciferol. Texila International Journal of Public Health 2025, 13, doi:10.21522/TIJPH.2013.13.02.Art092.
80. Schmidt, F.; Lapatki, B.G. Effect of variable periodontal ligament thickness and its non-linear material properties on the location of a tooth's centre of resistance. Journal of Biomechanics 2019, 94, 211-218, doi:10.1016/j.jbiomech.2019.07.043.
81. Schuldt, L.; von Brandenstein, K.; Jacobs, C.; Symmank, J. Oleic acid-related anti-inflammatory effects in force-stressed PdL fibroblasts are mediated by H3 lysine acetylation associated with altered IL10 expression. Epigenetics 2022, 17, 1892-1904, doi:10.1080/15592294.2022.2090654.
82. Sharma, S.; Patil, A.; Biyani, S.; Swarnkar, S.; Gera, M. Unveiling molecular dynamics: the effects of functional mandibular advancement and a one-month recovery period on myostatin and myosin-1c gene expression in masticatory muscles of young male Wistar rats. Folia Medica 2026, 68, doi:10.3897/folmed.68.e169836.
83. Smith, R.J.; Burstone, C.J. Mechanics of tooth movement. American Journal of Orthodontics 1984, 85, 294-307, doi:10.1016/0002-9416(84)90187-8.
84. Sobhani, M.; Bathaei, Y.; Afzalan, A.; Hormozi, S.; Shanehsaz, Z.; Nabizadeh, S.; Gashtasb, K. Biological Interplay between Orthodontic Forces and Apical Papilla-derived Stem Cells: A Review on Mechanisms and Implications. Galen Med J 2025, 14, e3946, doi:10.31661/gmj.vi.3946.
85. Steinberg, T.; Jung, B.; Husari, A.; Bai, S.; Tomakidi, P. Shaping Orthodontics of the Future: Concepts and Implications from a Cellular and Molecular Perspective. International Journal of Molecular Sciences 2025, 26, doi:10.3390/ijms26178203.
86. Tang, Z.; Yang, Y. Mechanobiology in orthodontic tooth movement: update on knowledge and clinical implications. Seminars in Orthodontics 2026, 32, 338-347, doi:10.1053/j.sodo.2025.10.009.
87. Utreja, A. Low-Magnitude Forces for Bone Modeling and Remodeling in Dentofacial Orthopedics. Current Osteoporosis Reports 2018, 16, 277-282, doi:10.1007/s11914-018-0437-9.

88. van Dijk, P.J.; Alvarez, C.M.; Desta, H.T. Interdisciplinary Orthodontic–Periodontal Management in Adult Patients: Timing, Sequencing, and Risk Control. Asian Journal of Periodontics and Orthodontics 2025, 5, 246-256, doi:10.51847/GrVLqrok4r.
89. Vora, S.R.; Bianchi, J.; Frazier-Bowers, S.A.; Lamani, E.; Akyalcin, S.; Kapila, S. Preface to the 10th Biennial COAST Conference: AI- and Biomedicine-Driven Precision Orthodontics and Craniofacial Care. Orthodontics & Craniofacial Research 2025, 28, S4-S9, doi:10.1111/ocr.70079.
90. Wang, Y.F.; Yu, S.L.; Zhang, Y.B.; Yang, X.L.; OuYang, T.; Shi, G.P.; Chen, L.L. Biological Determinants and non-pharmacological management of orthodontic pain. Iscience 2026, 29, doi:10.1016/j.isci.2026.116172.
91. Weber, A.K.; Torres, E.P.; Ismail, N.S. Integrating Periodontal Phenotype in Orthodontic Force and Timing: A Framework. Asian Journal of Periodontics and Orthodontics 2025, 5, 277-287, doi:10.51847/pCcyVYVizC.
92. Yadalam, P.K.; Joyson, J.J.; Mani, E.; Ayyachamy, S.; Mayurika, S.R. AI-Driven Stratification of Gingival Inflammation Progression During Orthodontic Aligner Therapy: A Dynamic Modeling Approach. In Proceedings of the Proceedings of the 2026 12th International Conference on Communication and Signal Processing, ICCSP 2026, 2026; pp. 830-835.
93. Yassir, Y.A.; McIntyre, G.T.; Bearn, D.R. Orthodontic treatment and root resorption: an overview of systematic reviews. *European Journal of Orthodontics* **2021**, *43*, 442-456, doi:10.1093/ejo/cjaa058.
94. Zhang, J.; Sun, B.; Zhao, H.; Zhang, T.; Chen, F.; Lin, J. Analysis of salivany peptidomics during orthodontic treatment. Chinese Journal of Orthodontics 2020, 27, 45-48, doi:10.3760/cma.j.cn115797-20191202-20110.
95. Zhang, M.; Ning, N.; Hong, Y.; Zhou, M.; Gong, X.; Zeng, L.; Wu, Y.; Ye, H.; Kang, T.; Chen, X. Digital working process in diagnosis, treatment planning and fabrication of personalized orthodontic appliances. Digital Medicine 2023, 9, doi:10.1097/DM-2023-00004.
96. Zhou, Y.K.; Zhang, G.M.; He, H. Mechanical forces orchestrate the epigenetic landscape of oral mesenchymal stem/progenitor cell fate in dental and periodontal tissues. *Frontiers in Cell and Developmental Biology* **2026**, *14*, doi:10.3389/fcell.2026.1743397.
